# Investigating attention mechanisms for plant disease identification in challenging environments

**DOI:** 10.1016/j.heliyon.2024.e29802

**Published:** 2024-04-17

**Authors:** Sangeeta Duhan, Preeti Gulia, Nasib Singh Gill, Piyush Kumar Shukla, Surbhi Bhatia Khan, Ahlam Almusharraf, Norah Alkhaldi

**Affiliations:** a Department of Computer Science & Applications, Maharshi Dayanand University, Rohtak, 124001, Haryana, India; b Computer Science & Engineering Department, University Institute of Technology, Rajiv Gandhi Proudyogiki Vishwavidyalaya (Technological University of Madhya Pradesh), Bhopal, Madhya Pradesh, India; c Department of Data Science, School of Science, Engineering and Environment, University of Salford, UK; d Department of Electrical and Computer Engineering, Lebanese American University, Byblos, Lebanon; e Department of Business Administration, College of Business and Administration, Princess Nourah Bint Abdulrahman University, P.O. Box 84428, Riyadh, 11671, Saudi Arabia; f Department of Computer Science, School of Computer Science and Information Technology, King Faisal University, Saudi Arabia

**Keywords:** Attention mechanism, Computer vision, Deep learning, Classification, Plant disease detection

## Abstract

There is an increasing demand for efficient and precise plant disease detection methods that can quickly identify disease outbreaks. For this, researchers have developed various machine learning and image processing techniques. However, real-field images present challenges due to complex backgrounds, similarities between different disease symptoms, and the need to detect multiple diseases simultaneously. These obstacles hinder the development of a reliable classification model. The attention mechanisms emerge as a critical factor in enhancing the robustness of classification models by selectively focusing on relevant regions or features within infected regions in an image. This paper provides details about various types of attention mechanisms and explores the utilization of these techniques for the machine learning solutions created by researchers for image segmentation, feature extraction, object detection, and classification for efficient plant disease identification. Experiments are conducted on three models: MobileNetV2, EfficientNetV2, and ShuffleNetV2, to assess the effectiveness of attention modules. For this, Squeeze and Excitation layers, the Convolutional Block Attention Module, and transformer modules have been integrated into these models, and their performance has been evaluated using different metrics. The outcomes show that adding attention modules enhances the original models' functionality.

## 1 Introduction

The global agriculture industry is facing significant challenges due to economic, environmental, and demographic pressures. With the growing global population, increasing agricultural production is necessary. However, achieving this goal is not easy. In this context, India stands out as a prominent player in the agricultural sector. It holds the second position in the production of essential crops such as sugarcane, rice, cotton, wheat, fruits, tea, and vegetables. Furthermore, around 60 % of India's workforce is employed in agriculture, contributing 17 % to the nation's GDP (Gross domestic product). Despite these advantages, Indian agriculture struggles with low output, lagging behind that of other developing countries by 30-5%. This poses a significant challenge for the country. Factors such as pest and disease outbreaks, soil fertility deficiencies, insufficient water availability, and the impact of climate change are all responsible for the stagnation of agricultural productivity in India. Among these challenges, plant diseases, particularly fungal pathogens, emerge as a critical concern and a major limiting factor for crop yields [1]. To tackle the aforementioned challenges, the integration of machine learning and attention mechanisms holds great promise for effective plant disease identification. Machine learning (ML) and Deep Learning (DL) methods can be leveraged, to quickly and precisely identify agricultural diseases by analysing images of various plant components such as leaves, stems, flowers, and fruits. Among these components, leaves have become a widely adopted focal point for disease identification, as most disease symptoms prominently manifest on them. Accurately identifying plant diseases is difficult because real-field images often contain complex backgrounds, the potential for plant leaves to be obscured by other plant parts, and the occurrence of several diseases on a leaf picture. In this scenarios, the incorporation of machine learning algorithms featuring attention mechanisms becomes crucial in overcoming these obstacles and improving disease recognition accuracy.

Attention mechanisms allow models to concentrate on particular segments of an image that are more informative for disease identification. By assigning different weights or attention scores to various parts of an image, these mechanisms enable the model to prioritize disease-related features and disregard irrelevant or misleading information. Selective attention has the potential to improve the precision of categorizing diseases, even when several diseases share similar symptoms. The attention mechanism draws inspiration from the way our biological systems function, as they prioritize unique characteristics when confronted with a lot of information. Since the advent of deep neural networks, several different application domains have made extensive use of attention mechanisms [2].

Attention mechanisms have proven successful across diverse fields like computer vision, speech recognition, and natural language processing. In the realm of computer vision, attention mechanisms play a crucial role by selectively focusing on relevant regions within an image, assisting networks to arrive at better decisions. The use of attention mechanisms has the potential to improve a variety of applications, including semantic segmentation, classification, object recognition, image captioning, 3D vision, and super resolution. The primary goal of this study is to investigate the various attention mechanisms that are used at different stages of identifying plant diseases and figure out how they help with feature extraction, which in turn makes disease recognition better. It is imperative to investigate the application of attention mechanisms in plant disease recognition, given their potential benefits in improving model performance and interpretability. This research attempts to provide a comprehensive analysis of attention mechanisms and how they affect different methods for identifying plant diseases.

This study's main contribution.

• This work offers a comprehensive review of different attention mechanisms, categorizing them based on the input they consider, the level of granularity they operate on, and the methods used to calculate attention weights.
• This study focuses on the diverse attention mechanisms employed by researchers to efficiently identify plant diseases. To the extent of our understanding, there is presently an absence of any other research investigation that specifically examines the attention mechanisms employed in plant disease detection.
• This study provides an in-depth survey of research that utilizes attention mechanisms to improve the performance of various tasks like image segmentation, feature extraction, object detection, and classification that are involved in developing plant disease recognition systems.
• This study presents a detailed specification of the type of network design, loss function, and deep learning framework used by researchers to create solutions for plant disease identification.
• This work performs a comparative analysis to analyse the effect of attention mechanisms on DL models. Various attention mechanisms are integrated into state-of-the-art models, including ShuffleNetV2, EfficientNetV2, and MobileNetV2, to evaluate their effectiveness.

This study is categorized into six sections, each serving a specific purpose. The initial section serves as an introduction, providing an overview of the main motivation and objectives of the study. Subsequently, in the second section, we discuss various attention mechanisms and their categorization. The third section explores different types of attentional mechanisms. In the fourth section, various studies proposed by researchers are examined, concentrating on leveraging attention mechanisms to improve the model's performance for efficient plant disease identification. Additionally, the fifth section conducts a comparative analysis between three deep learning models, both with and without the various attention modules. A discussion about the future potential of these mechanisms and the remaining challenges associated with their utilization is given. The study conclusion is presented in Section 6.

## 2 Related work

This section includes the recent studies that have reviewed various attention mechanism. Study [3], conducted a thorough examination on attention mechanisms for computer vision task and categorized them based on their approach, encompassing temporal attention, channel attention, branch attention, spatial attention, and various combinations of these methods. The main focus of this study was to categories attention approaches according to their data domain rather than their specific application area.

The authors of [4], classified image super-resolution models into different categories based on their network design, such as residual, dense, convolution, attention, distillation, and extremely lightweight solutions. This study's primary objective was to investigate lightweight approaches for image super-resolution that make use of deep learning techniques and attention mechanisms. This review serves as a valuable framework for our own study, as it provides us with a structured approach to follow.

In [5], details about the attention mechanisms used with neural networks is given, exploring their origins and recent advancements. The researchers provide comprehensive insights into various variants of attention models, including transformer and self-attention. Study [6], conducted a comprehensive analysis of the attention mechanism in deep learning, encompassing its key methodologies, practical implementations, diverse applications, and potential avenues for future development. This paper also describes how attention models are used to improve performance and what application domains can benefit from attention mechanisms.

Building upon this existing literature, our study contributes by categorizing attention mechanisms based on input nature, level of detail, and attention weight calculation method. A comprehensive examination of the attention mechanisms utilized in recent studies of plant disease identification for extracting relevant features at various stages, including segmentation, feature extraction, object identification, and classification, is done. This research conducted a comparative analysis by examining DL models, with or without attention modules to see the effectiveness of attention mechanisms on models. Furthermore, the paper addresses the current challenges of incorporating attention mechanisms into deep learning and explores potential future directions. It is worth noting that, to our knowledge, there is no existing literature review that delves as deeply into attention mechanisms for plant disease recognition, highlighting the significance of our study in advancing this field.

## 3 Understanding attention mechanisms

A significant advancement in deep learning, attention mechanisms revolutionize how models process information, enabling them to concentrate on specific features within a dataset. Modelled after human cognition, these mechanisms dynamically weigh various input components based on their importance, allowing the model to prioritize relevant information while disregarding irrelevant details. Notably, attention mechanisms are recognized for their ability to enhance model accuracy by focusing on essential information and filtering out noise. This capability is particularly advantageous in the context of plant disease identification, where precision is paramount. By selectively directing attention to specific regions or features within plant images, these mechanisms enable more precise and nuanced disease detection, resulting in more accurate diagnoses [7].

Moreover, attention mechanisms contribute to interpretability by highlighting which sections of an image are most influential in the classification process. This transparency provides valuable insights into how the model generates predictions, facilitating model validation and refinement. Additionally, attention mechanisms are highly adaptable and seamlessly integrate into a variety of deep learning architectures commonly used in plant disease detection, such as convolutional neural networks (CNNs) and Transformer models. This adaptability allows researchers to tailor models to specific task requirements and optimize performance across diverse datasets and plant species, underscoring their significance in advancing automated plant disease detection.

These mechanisms are classified based on a variety of factors, including the type of information they receive, such as spatial attention, which focuses on specific areas of an image, or channel attention, which improves CNN feature representations by highlighting specific channels or feature maps in the input. Furthermore, the granularity at which attention mechanisms operate varies, with global attention considering the entire input and local attention focusing on specific regions of interest within the input. Attention mechanisms differ in the process used to calculate attention weights Self-attention mechanisms calculate attention scores by examining the correlations between every pair of elements in the feature map or input sequence, whereas cross-attention mechanisms compute attention scores among elements from different input sequences or modalities and then use these scores to weight the information shared between them. Understanding these categorizations allows researchers to effectively use attention mechanisms to improve the performance of DL models. This study focuses on categorizing and describing the characteristics of these attention mechanisms.

### 3.1 Type of input considered

#### 3.1.1 Channel attention

**Fig. 1:**
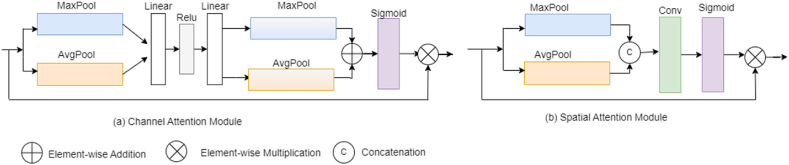
(a) Cam (b) SAM [11].

Attention mechanisms have distinct approaches tailored to different types of input considerations. One such method involves using channel information as input for attention mechanisms. In this paradigm, attention mechanisms refine feature representations within Convolutional Neural Networks (CNNs) by emphasizing specific channels or feature maps that are deemed critical for the task at hand. An image is represented by three channels: R, G, and B, which represent the red, green, and blue intensity levels of each pixel in the image. Through convolutional operations, each channel generates new channels with distinct information. By assigning weights to each channel to indicate its relevance to key information, a higher weight signifies greater relevance, thereby highlighting the importance of that channel [8]. Channel attention module (CAM) look for interactions and dependencies among various feature representations across multiple channels. The “squeeze-and-excitation” approach by Ref. [9] is utilized in CAM. The “squeeze” process reduced the dimensions of feature maps to just a single value (i.e., channel descriptor) by applying average pooling or max pooling. Fig. 1 (a) depicts the CAM [10]. The input tensor is processed initially in the CAM by an adaptive average pooling layer. This layer reduces the input tensor's spatial dimensions to 1x1 while keeping the channel dimension. The output is subsequently sent to a linear layer, which decrease the channel dimension by a predetermined reduction ratio. The rectified linear unit (ReLU) activation function is used for bringing nonlinearity into the network. Following that, another linear transformation is applied to restore the original channel dimension. To confine values within the range of 0–1, the sigmoid activation function is utilized, producing channel attention weights. These weights are then multiplied element by element with the input tensor so as to emphasize or minimize the importance of individual channels.

The Shuffle Attention technique focuses on channel-wise attention, which involves dividing input channels into groups and creating attention maps to highlight relevant aspects within each group. Shuffle attention initially splits the input feature map into several groups and simultaneously processes the sub-features within each group. Sub-features are combined, the outcomes from both branches are concatenated, and a channel shuffle operation makes it easier for various sub-features to communicate with one another. It has demonstrated improved performance in tasks like as instance segmentation, and object identification by including proposed shuffle attention module into different CNN models [12].

#### 3.1.2 Spatial attention

The spatial attention technique prioritizes spatial information for feature enhancement and can be applied at various scales, allowing the model to focus on both detailed information and broader contextual areas. By adaptively attending to different region of the input, the model can understand variations in scale, and appearance, leading to improved performance on visual tasks. As presented in Fig. 1 (b), the first step in the spatial attention module (SAM) [10] includes processing the input tensor via separate max pooling and min pooling operations along the channel dimension. The max pooling operation identifies the maximum values across channels, while the min pooling operation identifies the minimum values. These outcomes are then combined along the channel dimension, resulting in a tensor comprising two channels. Subsequently, a 2D convolution operation is employed to manipulate the tensor and generate a single-channel output. Following that, the sigmoid activation function is employed to compress the values between 0 and 1. These values represent the spatial attention weights, which measure the importance of specific spatial regions. Finally, the attention weights are element-wise multiplied with the input tensor, emphasizing or downplaying specific spatial regions based on their relevance.

The term “soft attention” or “soft attention mechanism” refers to one particular type of spatial attention mechanism. In this approach, model takes a feature map as input, and generates attention weights for each spatial point. The attention weights represent the significance or relevance of each regions, allowing the model to emphasize or de-emphasize certain regions during computation. Soft attention is introduced by Ref. [13]. The soft attention mechanism employed an independent neural network module to produce attention weights. These weights were subsequently utilized to calculate a weighted sum of image features. By training the model, the attention weights were learned, enabling automatic identification of the most pertinent image regions for generating precise and meaningful captions.

Coordinate attention is a type of spatial attention mechanism. Unlike typical channel attention mechanisms such as SENet [9], which only capture inter-channel information, coordinate attention includes spatial information as well as channel interactions. This technique captures long-range dependency along one spatial dimension while keeping precise positioning information along the other [14].

#### 3.1.3 Mixed attention

**Fig. 2:**
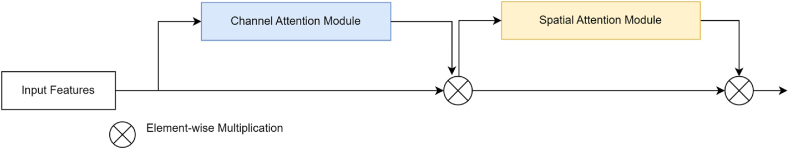
CBAM module.

The mixed attention mechanism refers to a type of attention mechanism that combines different attention mechanisms or components within a neural network model. It involves the integration of multiple sources of information obtained from utilizing diverse attention mechanisms, such as spatial attention, channel attention, or other variants, for enhancing the model's ability to collect diverse information. The attention weights were learned during the training process. In Ref. [15], Dual Attention Network (DAN) is proposed which combine channel and spatial attention mechanism for scene segmentation. In Ref. [10], Convolutional Block Attention Module (CBAM) is proposed which contain two sub modules CAM and SAM as shown in Fig. 2. For better feature refinement, CBAM can be incorporated into any CNN architecture because of their lightweight structure.

### 3.2 Method of computing attention weights

#### 3.2.1 Self attention

Various attention mechanisms use different approaches to calculate attention weights, which are adapted to their specific purposes and input characteristics. Self-attention mechanisms, for example, compute attention weights by examining the associations between each pair of components in an input sequence or feature map. The self-attention mechanism enables interaction between elements of input ("self”) and establishes the level of attention that should be given to each element. The resulting outputs are a combination of these interactions and corresponding attention scores. By assigning attention weights to each pixel or region in the self-attention layers, the model may dynamically learn to emphasize the most relevant features of the input images. This feature allows the model to focus on locations having the most relevant information for discriminating between healthy and unhealthy plants.

#### 3.2.2 Cross attention

In deep learning systems, cross-attention, often referred to as cross-modal attention or inter-attention, is a method that facilitates communication and interaction across several modalities or representations within a model. With cross-attention, a model can analyse data from one sequence or modality while attending to aspects from another, in contrast to self-attention, which concentrates on capturing dependencies inside a single sequence or feature set. When dealing with tasks that involve pairs of sequences, like machine translation, this variation of attention is very helpful since it helps the model identify the links between elements in various sequences [16]. A dual-branch transformer model named CrossVit is proposed in Ref. [17], for multi-scale feature learning in image classification. In order to maintain linear computing cost while enabling effective information flow between small-patch and large-patch tokens, cross-attention is utilized as an effective fusion technique. Cross attention is used in the U-Transformer network [18] for medical image segmentation to improve the capacity of the U-Net decoder to recover spatial information from skip connections. Cross-attention is used to filter out noisy or unnecessary regions from the skip connection features, so that the model can concentrate on semantically rich areas for precise segmentation.

#### 3.2.3 Transformer based attention

**Fig. 3:**
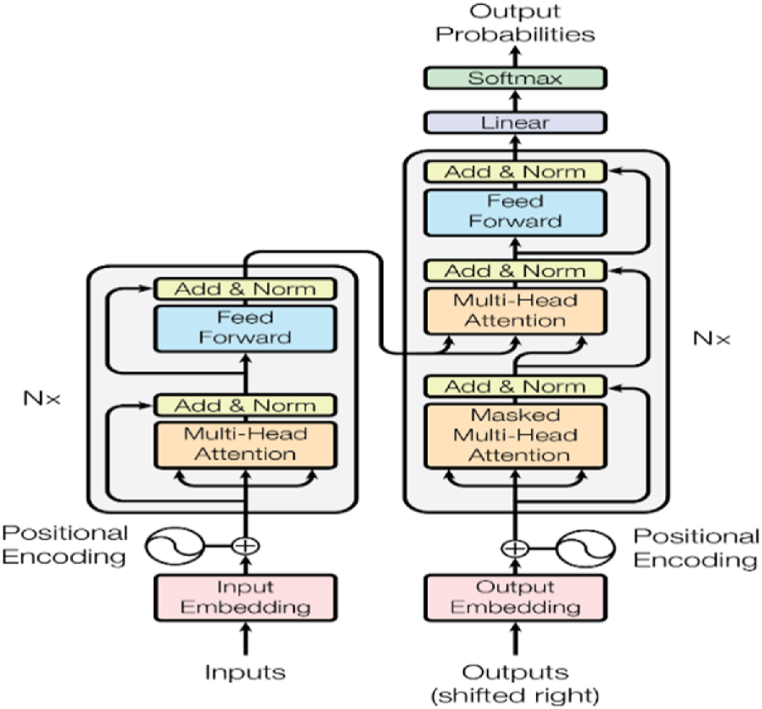
Transformer architecture [11].

In 2017, study [11] achieved a significant advancement with the creation of a ground-breaking neural network known as the “Transformer.” The Transformer, which was first created for NLP tasks, revealed to the world the amazing powers of self-attention processes. By utilizing self-attention, this design enables models to process sequences in parallel, effectively capturing long-range dependencies. The transformer model follows an encoder-decoder architecture, where multiple encoders and decoders are stacked (as represented by Nx in Fig. 3). This stacking implies that the outcome of one encoder becomes the input for the subsequent encoder, and similarly, the outcome of one decoder becomes the input for the adjacent decoder [19]. According to this approach, the encoder receives a series of symbols as input, and the decoder produces a series of symbols, one element at a time, as the output [11]. The transformer model relies mainly on the attention function to produce output, it maps a query and a set of key-value pairs.

Specifically, the attention method works on a set of queries that are arranged in a matrix Q. Similarly, the values and keys are organized into matrices V and K, respectively. The resulting matrix of outputs is computed through a specific process as shown in Eq. (1):

(1) $$Attention\left(Q,K,V\right)=VSoftmax\left(\frac{QK^{T}}{\surd d_{k}}\right)$$

Where, $d_{k}$ is keys of dimensions.

#### 3.2.4 Multi-head attention

**Fig. 4:**
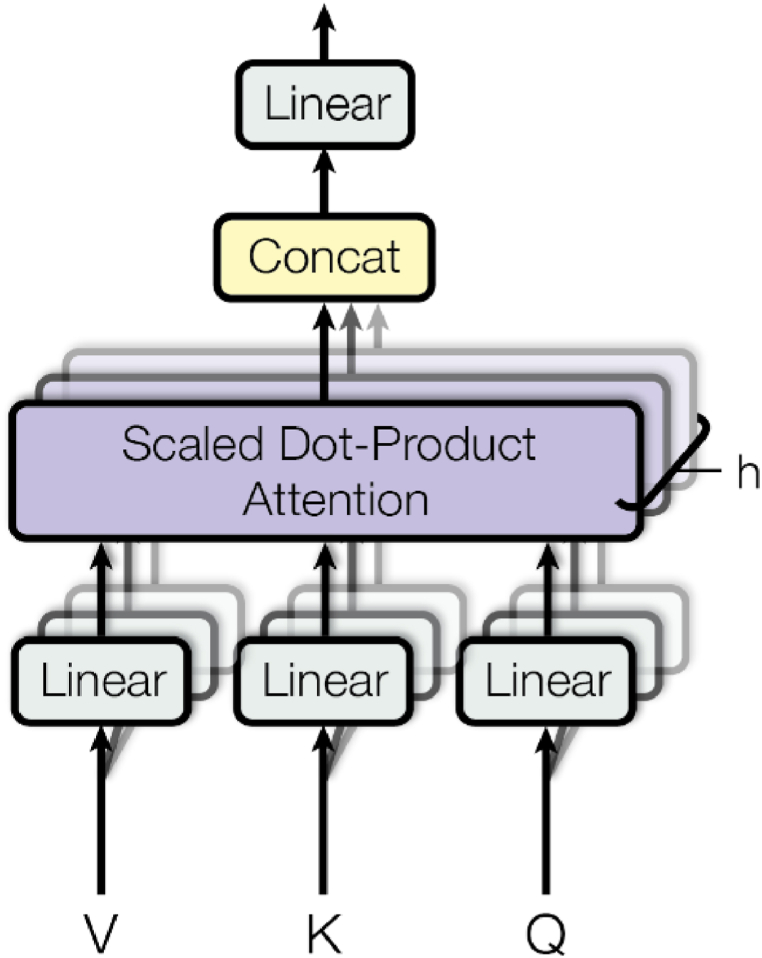
Multi-Head Attention [11].

Multi-head attention, as seen in Fig. 4, allows the model to collectively focus on input from distinct representation subspaces and positions. In Ref. [11], researchers begin by applying a linear transformation to the input matrices Q, K, and V, then compute attention as in Eq. (2):

(2) $$Attention\left(w^{Q}Q,W^{K}K,W^{V}V\right)=W^{V}VSoftmax\left(\frac{W^{Q}QW^{K}K^{T}}{\sqrt{d_{k}}}\right)$$

Where, $W^{K}$ , $W^{Q}$ , $W^{V}$ are all learnable parameters. In multi-head attention, linear transformation is applied to the matrices and perform attention on number of heads and then these heads are concatenated and then linear transformation is applied again as shown in Eqs. (3), (4):

(3) $$MultiHead\left(Q,K,V\right)=Concat\left({head}_{1},{head}_{2},\ldots {head}_{i}\right)w^{o}$$

Where,

(4) $${head}_{i}=Attention\left(QW_{i}^{Q},KW_{i}^{K},VW_{i}^{V}\right)$$

Transformer-based attention employs self-attention and multi-head attention to handle input sequences. In the transformer architecture, a self-attention layer first process the input sequence, then a feed-forward network, and finally another self-attention layer. This sequence of layers is repeated multiple times, with residual connections and layer normalization applied between each layer. Transformer-based attention has been shown to be highly effective in modelling sequential and spatial dependencies in natural language and computer vision tasks [19].

In summary, self-attention serves as a foundational element within multi-head attention, which in turn functions as a critical component in the overall transformer-based attention mechanism. Some common transformer-based attention variants used in computer vision are: Vision Transformer (ViT) [20], Data-efficient Image Transformers (DeiT) [21], Swin Transformer [22], Pooling-based Vision Transformer (PiT) [23], and DEtection TRansformer (DETR) [24].

### 3.3 Level of granularity

Attention mechanisms work at multiple levels of granularity, allowing models to concentrate on different sections of the input data according to their specific needs. On a broader scale, attention processes can be categorized according to the amount of information they consider. Global attention techniques allow models to focus on every element in an input sequence or feature map. Local attention mechanisms, on the other hand, concentrate on particular areas or portions of the information, enabling more tailored processing while also lowering computing complexity. To improve discriminative features for classification, the Squeeze-and-Excitation Network (SENet) [9] for instance, uses global attention to recalibrate feature maps over the entire image based on channel-wise relationships [25].

#### 3.3.1 Multi-scale attention

Multi-scale attention refers to the model's capacity to pay attention to and capture information at multiple scales or levels of detail within an input. This is typically achieved by incorporating mechanisms that can aggregate features from different scales, such as using dilated convolutions, feature pyramid networks [26], or pyramid pooling modules [27]. The aim is to enable the model to grasp detailed as well as comprehensive contextual information, thereby enhancing its understanding of the input across various levels and scales. Example of multi-scale attention models are: Non-local Neural Networks [28], Deep Layer Aggregation (DLA) [29], Scale-Aware Trident Network [30], and Path Aggregation Network (PANet) [31].

In conclusion, attention mechanisms provide an adaptable framework for deep learning feature representation. Attention processes help models extract meaningful information and increase performance across a variety of tasks by carefully directing focus to key components within the input data. Importantly, various attention mechanisms are tailored to specific areas of data processing, with each providing distinct benefits based on the task requirements. However, it is important to note that attention processes are not mutually exclusive; rather, they can complement one another when utilized alone or in combination. For example, a task may benefit from the simultaneous use of global and local attention mechanisms to collect both global patterns and specific details within the data. This adaptability demonstrates attention mechanisms' versatility and usefulness in resolving a variety of machine learning problems in addition to their importance as a key tool in increasing model capabilities.

## 4 Attention mechanisms in plant disease identification

**Fig. 5:**
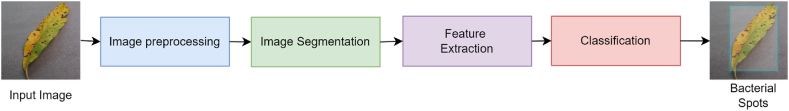
Steps involves in plant disease identification.

To create an automated system for identifying plant diseases, various stages must be undertaken, as depicted in Fig. 5. The initial stage involves capturing images of plants from real fields. While diseases can occur in various plant parts, such as roots, stems, or leaves, leaf images are commonly used for disease identification since most visible symptoms appear on leaves. Once the images are obtained, the subsequent stage involves image pre-processing. This entails resizing the images to suit the model's requirements and applying denoising techniques to eliminate distortions. Furthermore, image augmentation techniques like scaling, flipping, rotation, translation, cropping, and deep learning augmentation methods such as GANs are employed to augment the dataset, for better training of the data-hungry ML model.

In the third stage, image segmentation is performed to identify regions of interest (ROIs) where infected areas are present in the image. Subsequently, in the fourth stage, feature extraction techniques are applied to extract relevant features from the images, which facilitate the ML model in identifying diseased or healthy regions. The final stage involves classification, where the disease class affecting the plant leaf is determined. The output obtain from this stage is the probability values associated with different types of diseases that could be present on the plant leaf. To enhance the plant disease identification system, attention mechanisms are integrated into the segmentation, feature extraction, object detection, and classification phases. This enables the models to allocate more attention or resources to crucial regions of an image while reducing emphasis on less relevant regions. This section focuses on the techniques and models proposed by researchers that utilize attention mechanisms to develop solutions for plant disease detection and classification. Detailed information regarding pre-processing, augmentation, segmentation, and classification techniques employed in recent studies, as well as the frameworks utilized and the achieved accuracy, can be found in Table 1, which provides a comprehensive summary of various plant disease recognition studies.

Attention mechanisms, known for their versatility, provide crucial support at several phases of plant disease identification. In image segmentation tasks, mechanisms such as spatial attention or multi-scale attention that target specific regions of interest within plant images, allowing for more exact demarcation of diseased areas. In the context of feature extraction, self-attention mechanisms prove particularly effective at identifying complex patterns and correlations in the data, which makes it easier to extract distinguishing traits linked to distinct diseases. In object detection, both channel and spatial attention mechanisms are essential because they direct focus to disease-related anomalies and allow for the identification of affected areas within complex plant leaf images. Finally, in the classification step, mechanisms like as global attention can be used to emphasize informative portions of the image, resulting in more accurate disease classification based on visual characteristics. By leveraging on these various attention mechanisms at different phases, systems for identifying plant diseases can attain enhanced precision, resilience, and effectiveness, thereby making a substantial contribution to the advancement of agricultural sustainability and crop management.

**Fig. 6:**
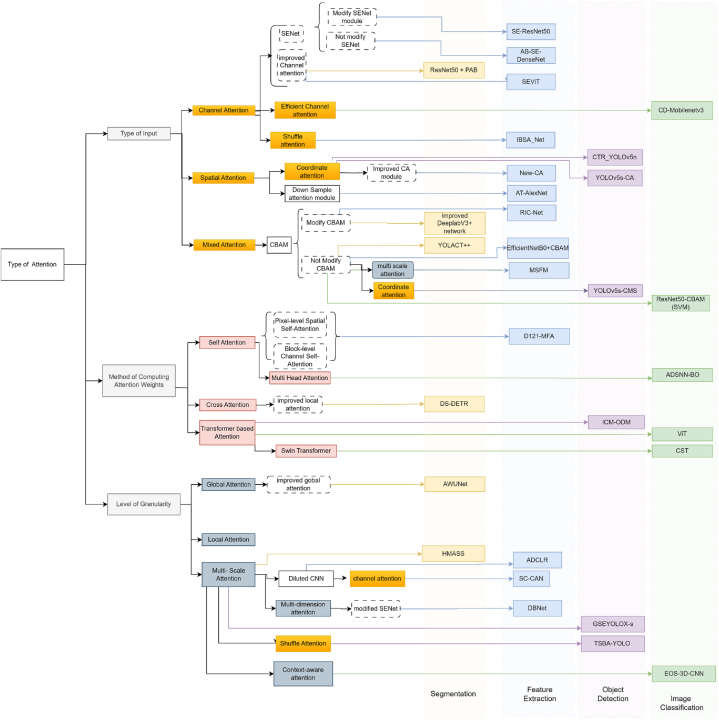
Diagrammatic representation of various attention mechanism along with the studies that employ these mechanisms

In this study, various techniques proposed by researchers are reviewed, which utilize attention mechanisms to enhance different aspects of plant disease identification, including segmentation, feature extraction, classification, and object detection. Throughout the analysis, a spectrum of approaches is observed, ranging from the direct incorporation of existing attention mechanisms to innovative modifications tailored to specific plant disease identification tasks. Fig. 6 is presented to offer a succinct overview of the studies included in the analysis. This figure categorizes the techniques based on the stages of the plant disease identification process they target, facilitating a detailed examination of the attention mechanisms employed across segmentation, feature extraction, classification, and object detection. By scrutinizing Fig. 6, readers can discern the specific attention mechanisms utilized to enhance segmentation processes, improve feature extraction, refine classification accuracy, and enhance object detection in plant disease identification scenarios.

In this section, we delve into the intricacies of attention mechanisms utilized by researchers to enhance various facets of plant disease identification. Our focus lies predominantly on elucidating the methodologies where attention mechanisms are harnessed to bolster different tasks inherent in this domain. The ensuing figure, presented within this section, serves as a comprehensive guide, delineating the strategic integration of these mechanisms within the model architecture and delineating the specific attention modules employed. Through this detailed analysis, we aim to provide clarity on the nuanced applications of attention mechanisms, shedding light on their pivotal role in advancing the field of plant disease identification.

### 4.1 Leveraging attention mechanisms for image segmentation

Image segmentation is the task of dividing an image into semantically relevant areas. Attention mechanisms have evolved as an efficient strategy for improving image segmentation performance because they selectively focus on important image features. In recent studies, several attention processes have been used to extract relevant characteristics and improve segmentation task performance. In recent studies, various attention mechanisms have been utilized to extract relevant features that improve the performance of segmentation tasks. Some of these studies in the area of plant disease identification are discussed in this section.

#### 4.1.1 Improved DeeplabV3+ network

**Fig. 7:**
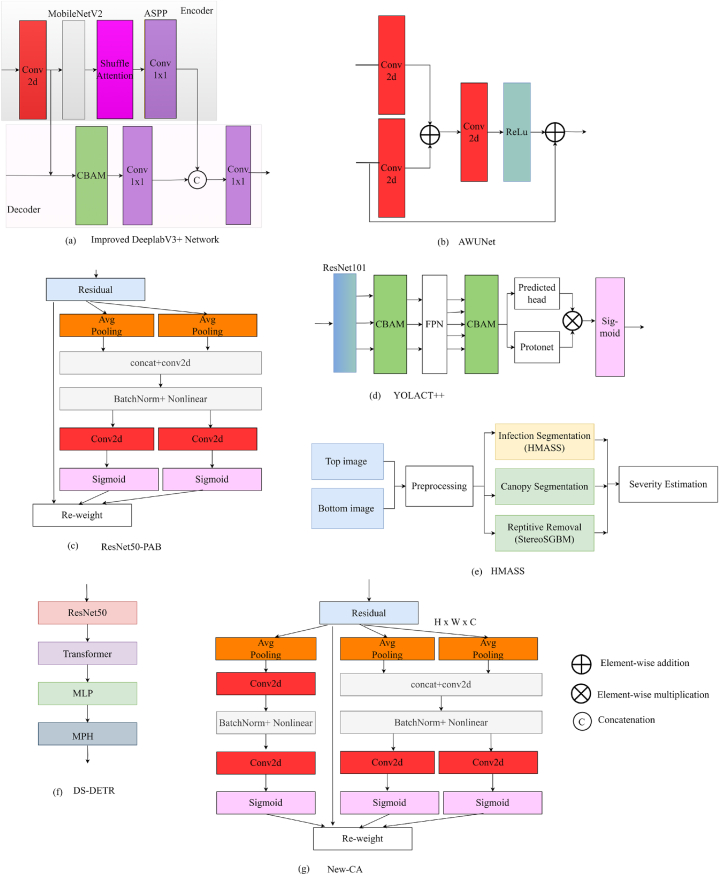
Network design of studies that focus on image segmentation utilizing attention mechanisms (a) Improved DeeplabV3+ Network; (b) AWUNet; (c) ResNet50-PAB; (d) YOLACT++; (e) HMASS; (f) DS-DETR; (g) New-CA

For better image segmentation, [32], suggested an improved DeeplabV3+ network. The encoder component, shown in Fig. 7 (a), employs MobileNetV2 [33] as the primary network for feature extraction instead of Xception network, which minimises computation and enhances speed. To handle sub-features, the shuffle attention module is utilized, while Atrous Spatial Pyramid Pooling (ASPP) [34] is used to fuse image context information to obtain high-level information and pass it to the decoder section. In the decoder, CBAM [10] is incorporated to enhance segmentation accuracy. Furthermore, to assign different weights for background and disease spots, a weighted loss function is utilized to ensure differential treatment during the training process, which is specified in Eq. (5):

(5) $$L=-\frac{1}{N}\sum_{i=1}^{N}\sum_{j=1}^{C}W_{j}t_{j}^{i}I_{n}\left(y_{j}^{i}\right)$$

Here, total pixel count is denoted by N, and total number of categories by C, i is the index representing a specific training pixel, j is the index denoting the class of that training pixel, true disease spot category of the ith training pixel's annotation is denoted by $t_{j}^{i}$, and $y_{j}^{i}$ is the predicted disease spot category for the ith training pixel. Additionally, $W_{j}$ is a weight parameter for category j, calculated as, $\frac{\left(N-N_{j}\right)}{N}$ where Nj represents the pixel count for category j.

#### 4.1.2 AWUNet

Study [35], introduced AWUNet (Attention-gated Wavelet pooled UNet) is a unique variant of the U-Net architecture [18] that integrates wavelet pooling and attention-gated skip connections. Attention gate module utilized in study is shown in Fig. 7 (b). Attention gate module and remodel skip connections is used to decrease the dimension of the feature map, wavelet pooling is used in between convolution layers instead of max pooling. AWUNet model integrates global and local information from both encoder and decoder paths, leveraging learned attention weights to focus on salient features for improved semantic segmentation. The suggested approach was evaluated against various deep learning techniques including U-Net [18], Visual Geometry Group (VGG) [36], and ResNet [37]. Mean Square Propagation (RMSPROP) optimizes the weights of the AWUNET model, and Lecun Normal is used for the kernel initializer.

#### 4.1.3 ResNet50 + PAB

A novel lightweight position attention block (PAB) as shown in Fig. 7 (c) is proposed in Ref. [38] that breaks channel attention into one-dimensional feature encoding. The PAB can capture long-range dependencies along one spatial direction while keeping precise position information along the other by reducing global pooling into direction-specific encoding procedures. PAB can be embedded in existing networks such as MobileNet [39], VGG [36], and ResNet [37]. The generalization ability of the position attention block was tested by using object detection models like YOLOv3 [40], YOLOv5 [41], and semantic segmentation models like Mask RCNN [42].

#### 4.1.4 YOLACT++

For better segmentation and detection of diseased spots, YOLACT++ with the attention module is proposed in Ref. [43] The suggested model has five components: In the initial stage, ResNet101 [37] is used as a feature extraction network, and in the second stage, the CBAM attention module [10] is used. In the third stage, the Feature Pyramid Network (FPN) architecture [26] is utilized to get the feature maps, which are fed into the second CBAM as showcase in Fig. 7 (d). The fourth stage involves a segmentation network that utilizes a prediction head structure to enhance the speed of segmentation. The network receives five feature maps as input and accomplishes three objectives: predicting target classification, bounding box, and mask coefficients. Other components of the segmentation network utilize Protonet to generate prototype masks that match the original image's size. The final step involves image post-processing, which includes thresholding, cropping, and fast mask re-scoring.

#### 4.1.5 HMASS

For analyse grape foliar disease infection across multiple scales, the hierarchical multi-scale attention for semantic segmentation (HMASS) network [44] was used in Ref. [45] which uses a series of stereo images captured using a utility task vehicle (UTV) in the field. The proposed solution consists of four primary components: disease infection segmentation, canopy segmentation, image overlap removal, and infection severity estimation, as depicted in Fig. 7 (e). Canopy segmentation involved utilizing colour filtering techniques to generate canopy masks in the images. The module responsible for identifying and removing overlapping regions between successive photographs made use of depth and GPS data. The stereo semi-global block matching (StereoSGBM) approach is used to obtain depth data. Finally, the ratio of infected areas to canopy areas in non-repetitive picture regions was calculated to assess the severity of disease infections.

#### 4.1.6 DS-DETR

Authors of [46], proposed the Disease Segmentation Detection Transformer (DS-DETR) for enhancing the convergence speed, and to minimize the training epochs required, an unsupervised pretrained UP-DETR model [24] is proposed. In the DS-DSTR model, to get the feature sequence vectors, ResNet50 is used, as depicted in Fig. 7 (f). Feature extracted are fed into the transformer; in the encoder phase of the transformer, improved Relative Position Encoding is utilized to give more attention to local features; and in the decoder phase of the transformer, the Spatially Modulate Co-attention (SMCA) module [47] is employed to extract features from various spatial positions. Results from the decoder phase are inputted into the mask prediction heads (MPH) to accomplish pixel-level segmentation of the identified targets.

#### 4.1.7 New-CA

To address the issue of disease recognition in a complex background conditions study [48], use GrabCut algorithm to process the real field image and make the background black so that a similarity can be established between real time and the images used for training the model. A new coordinate attention (CA) block is proposed, as shown in Fig. 7 (g) that obtains long-range dependencies along two spatial directions and along channel directions as well, instead of CA that obtains information along H- and W-directions only. Additionally, channel pruning is used to reduce the size of the model, and fine-tuning is performed to account for the performance of the model after pruning. With the new CA, ResNet50 [37] and GhostNet [49] both perform well in terms of minimising the error and shrinking the model size.

Attention mechanisms allow the model to concentrate on important regions of the input image, enhancing image segmentation by accurately identifying objects and boundaries. They facilitate the selection of valuable features from different spatial locations, capturing intricate details in scenarios with diverse object scales, shapes, or appearances. However, the integration of attention mechanisms may lead to increased memory usage and longer inference times, particularly in resource-intensive or time-critical applications.

### 4.2 Leveraging attention mechanisms for feature extraction

Feature extraction refers to the process of obtaining meaningful and informative representations, also known as features, from raw input data. In the context of computer vision, feature extraction typically involves transforming images or image patches into lower-dimensional representations that capture relevant visual information. The utilization of attention mechanisms enhances the discriminative capability of the model, allowing it to prioritize informative regions or features. This is particularly advantageous for tasks that necessitate precise discrimination, such as object recognition or fine-grained classification. Studies that utilized various attention mechanisms for extracting relevant features are discussed here.

#### 4.2.1 SE-ResNet50

**Fig. 8:**
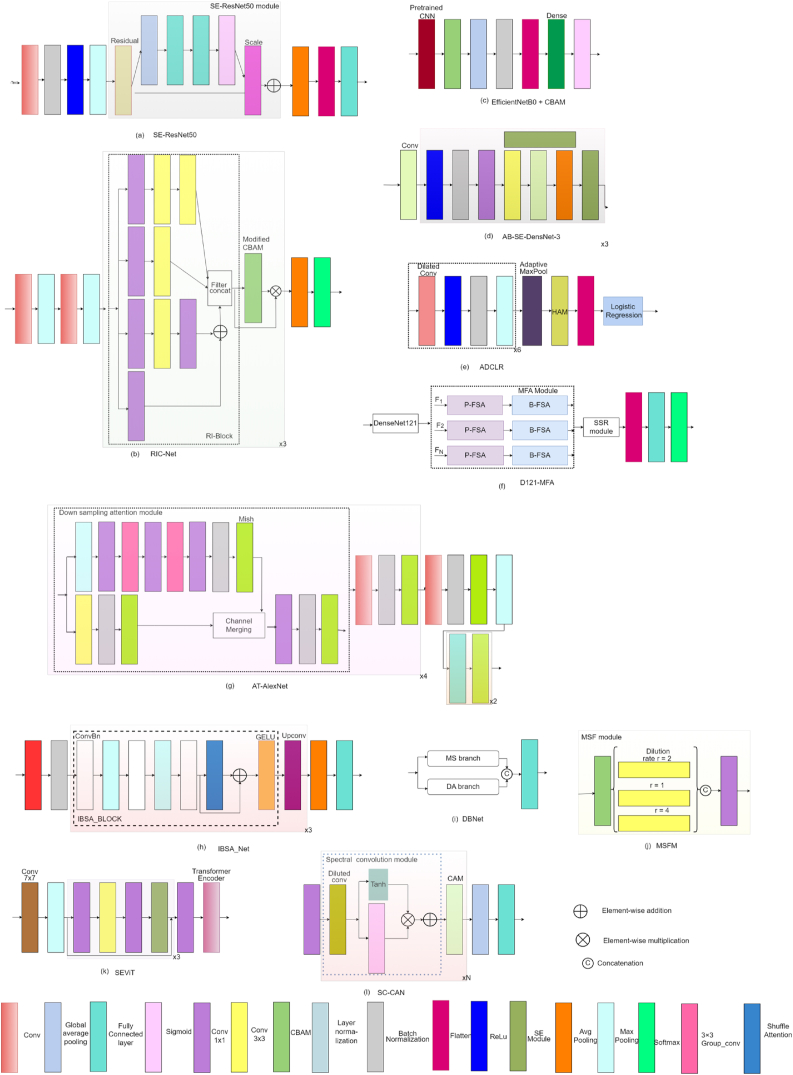
Network design of studies that focus on feature extraction utilizing attention mechanisms (a) SE-ResNet50; (b) RIC-Net; (c) EfficientNetB0 + CBAM; (d) AB-SE-DenseNet-3; (e) ADCLR; (f) D121-MFA; (g) AT-AlexNet; (h) IBSA_Net; (i) DBNet; (j) MSFM; (k) SEViT; (l) SC-CAN

A multi-scale CNN with residual blocks and Squeeze and Excitation (SE) module was introduced by Ref. [50] as shown in Fig. 8 (a) to identify tomato leaf diseases. The SE module recalibrates features by weighting feature channels by relevance. This recalibration approach improves the model's ability to extract complicated disease features by enhancing effective feature channels and reducing invalid ones. The SE module is directly integrated with the ResNet-50 architecture. This integration guarantees that the SE module works seamlessly with ResNet-50's existing layers, improving its feature extraction capabilities.

#### 4.2.2 RIC-Net

To reduce trainable parameters, RI-Block is proposed in Ref. [51] by merging the Inception structure with the residuals network. For accurate feature extraction, the CBAM module is introduced. Improvements are introduced in CBAM. In CAM, shared MLP's operations were initially replaced with two one-dimensional convolutions. Furthermore, a weighted operation was introduced to emphasize the significance of lesions. A fully trained RIC-Net model was then made available online to enable the real-time detection of plant diseases, as shown in Fig. 8 (b).

#### 4.2.3 EfficientNetB0+CBAM

To emphasize significant local regions and extract more distinctive features from the output feature map of a CNN, [52], employed the CBAM module, as depicted in Fig. 8 (c). The effectiveness of CBAM was demonstrated using pre-trained CNN models such as, MobileNetV2 [33], VGG19, ResNet50 [37], EfficientNetB0 [53], and InceptionV3 [54]. EfficientNetB0+CBAM has outperformed the original EfficientNetB0 model by giving the best accuracy.

#### 4.2.4 AB-SE-DenseNet

Study [55], propose an AB-SE-DenseNet model in which a DenseNet model [56] embedded with an SE module is used to extract useful features from global information and the AdaBound algorithm is used to accelerate model fitting and enhance the generalisation capability of the model. This study developed three different SE-DensetNet-1, SE-DensetNet-2, and SE-DensetNet-3 models that differ in how SE modules are embedded in the network. The SE-DensetNet-3 models, depicted in Fig. 8 (d), outperform other models by incorporating the SE module in both the transition layer and the dense block of DenseNet simultaneously.

#### 4.2.5 ADCLR

The authors of study [57] proposed attention-based dilated CNN logistic regression (ADCLR) as illustrated in Fig. 8 (e). For image pre-processing, colour space conversion is used to extract brightness and saturation levels, and normalization is used to reduce computation complexity. In order to address the issue of imbalanced data, synthetic images are created through the utilization of a technique known as Conditional Generative Adversarial Network (CGAN) [58]. In this paper, a new feature extraction approach is developed that uses dilated CNN with hierarchical attention mechanisms (HAM). Multiple hidden layers are used in dilated CNN for efficient learning of discriminatory features. And in the last for classification task, a logistic regression model was used.

#### 4.2.6 D121-MFA

The model proposed in Ref. [59] consists of four modules: First, to extract input image features, DenseNet121 [56] is used. Secondly, to obtain local and global features, a multi-granularity feature aggregation module (MFA) is developed that consists of two components: picture-level feature self-attention (P-FSA), which enables the extraction of discriminative features from various disease regions, and block-level feature self-attention (B-FSA), which improves the model's capacity to recognise the traits of various crop species. The MFA module collectively improves the feature aggregation process by incorporating attention mechanisms at different levels of granularity. Thirdly, to capture spatial geometric relationships between feature blocks, sequential spatial reasoning (SSR) is introduced, and in the last step, a classical classification head is used to categorize plant diseases as shown in Fig. 8 (f).

#### 4.2.7 AT-AlexNet

To improve model feature extraction capability and minimize information, [60], introduced a modified version of the AlexNet network [61] integrated with a sampling attention module, depicted in Fig. 8 (g). As a means of augmenting the network's non-linear expression capability, the Mish activation function is employed in place of ReLU. This substitution leads to a notable 0.65 % improvement in the model's recognition accuracy. Group convolution (GC) is utilized to decrease the trainable parameters and increase the diagonal correlation between the convolution kernels of adjacent layers.

#### 4.2.8 IBSA_Net

The authors [62], proposed the IBSA_Net model, which consists of IBMax_block, in which to reduce the number of parameters, an inverted bottleneck structure is used with batch normalization, and a MaxPool layer is also used in between ConvBN blocks, which are added to enhance the model's stability. To improve the capacity structure of the suggested model to obtain spatial location, the shuffle attention (SA) module is added together with residual and the GELU activation function, which is named IBSA_block. In the IBSA_Net model, as shown in Fig. 8 (h), there is the utilization of three IBSA_Blocks, with channel up-convolution (UPconv) employed between these modules. In the final block, global average pooling is applied to extract relevant information, followed by the FC layer.

#### 4.2.9 DBNet

The dual-branch network (DBNet) model suggested in Ref. [63] which consists of a multiscale joint branch (MS) and a multi-dimensional attention branch (DA) as illustrated in Fig. 8 (i). Both of the branch use VGG-16 as their backbone network. MS uses dilated convolution and asymmetric convolution kernels to obtain various receptive fields in parallel, which is useful when lesion information in images is scattered. To get information about the exact lesion location in the image, a novel attention mechanism called DA is utilized. In the DBNet, output obtained from the MS and DA branches is concatenated, and then the FC layer is introduced to get the output.

#### 4.2.10 MSFM

In [64], a lightweight multi-scale fusion model (MSFM) that contain EfficientNet-B6 [53] as the base network is introduced. The pre-trained network incorporates CBAM prior to each regularization stage to improve the model's capacity to choose features. A multi-scale fusion module is used to extract precise colour, texture, and context information from the image. CBAM is originally used in this module, as illustrated in Fig. 8 (j), to enhance the properties of tiny lesion information by learning relevant features. Then, dilated convolutions with various expansion rates are used to extract features.

#### 4.2.11 SEViT

A squeeze-and-excitation vision transformer (SEViT) model was developed in Ref. [65] for efficient detection of fine-grained and large-scale disease symptoms in plants and contains two modules. The first module is the pre-processing network, in which the ResNet101 model [37] is improved by embedded the SE module, which enhances disease features. The second module is the pretrained ViT model [20], which takes enhanced expression input from SE-ResNet101 and produces disease probability as illustrated in Fig. 8 (k). The developed model's limitations include large model parameters and a deep network. The severity level of the disease cannot be predicted; only the disease can be classified.

#### 4.2.12 SC-CAN

In [66], the Spectral Convolution and Channel Attention Network (SC-CAN) to distinguish between the spectral responses of stressed and healthy crops is introduced. The input to this network is given in the form of a sequence of spectral bands. To address the issue of class imbalance, the Synthetic Minority Oversampling Technique (SMOTE) is used to increase the sample size of the minority class. The SC-CAN comprises two components: the first is the spectral convolution module, which, to increase the receptive field, utilizes a dilated convolution layer with residual connections. This enables the extraction of global features even in shallow networks. The second module is the channel attention module, which takes refined feature maps obtained from dilated convolution layer as input and compute inter-channel relationships, as illustrated in Fig. 8 (l).

Attention mechanisms can be incorporated into various neural network architectures, such as recurrent neural networks (RNNs) and CNNs. They can complement existing architectures and improve their feature extraction capabilities. While embedding attention mechanisms improves feature extraction performance, they can make the interpretability of the learned features more challenging. Understanding which specific regions or features contribute to the model's decisions becomes more complex due to the attention mechanism's non-linear and implicit nature.

### 4.3 Leveraging attention mechanisms for object detection

Object detection is the process of identifying and locating objects in an image. However, this task can be quite difficult due to differences in how objects look, their size, and when they are partially obstructed. To tackle these challenges, attention mechanisms are employed in object detection models. The utilization of these mechanisms allows models to concentrate on the pertinent aspects of an image, adjust to objects with varying sizes, integrate contextual details, optimize resource allocation, and effectively manage complex scenes. By utilizing attention mechanisms, the models can significantly enhance their accuracy in detecting and localizing objects in complex visual environments. Below are a few examples of models that have been created by researchers, leveraging attention mechanisms to enhance the ability to identify diseases.

#### 4.3.1 YOLOv5s-CMS

**Fig. 9:**
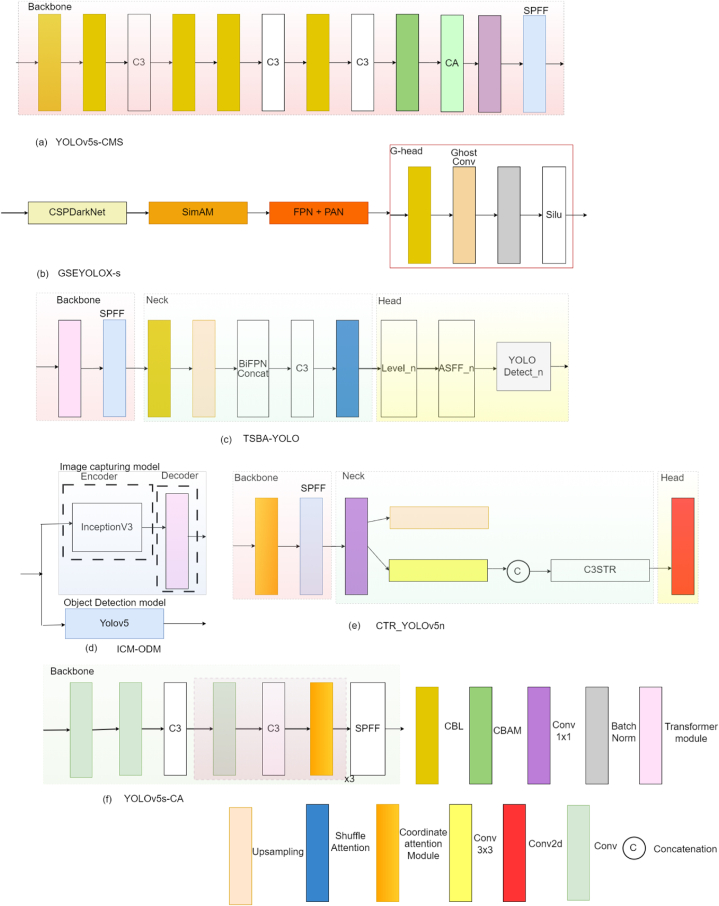
Network design of studies that focus on object detection utilizing attention mechanisms (a) YOLOv5s-CMS; (b) GSEYOLOX-s; (c) TSBA-YOLO; (d) ICM-ODM; (e) CTR_YOLOv5n; (f) YOLOv5s-CA

For detecting the root-knot nematode in cucumber plants, [67], used YOLOv5s [41] deep learning model. And to produce anchor boxes, k-means++ clustering algorithm was utilized. To identify key regions and capture distinguishable features from a small target, the dual attention mechanism CBAM and coordinated attention (CA) is embedded in the backbone of YOLOv5s as illustrated in Fig. 9 (a). The proposed model detects the affected region with a 3.1 % increase in mean average precision in comparison to the original model.

#### 4.3.2 GSEYOLOX-s

To determine the level of severity of Fusarium head blight (FHB) in wheat, [68], proposed a novel, lightweight model named GSEYOLOX-s. The proposed model is an improvement over YOLOX-s model. In the suggested model, a simple, parameter-free attention module (SimAM) [69] is included after the CSPDarknet backbone network of the original model so that the model concentrates on crucial components without increasing trainable parameters, and after the FPN [26] and PAN [31] structures, a G-head module is introduced to simplify the redundancy problem of feature maps to reduce complexity and increase speed as shown in Fig. 9 (b). In place of the IoU loss, the Efficient Intersection over Union (EIoU) Loss function is utilized to achieve a more precise localization of the disease area. GSEYOLOX-s reduces the parameters to 0.88 MB and increases the mean average precision (mAPa) to 2.52 % from the original YOLOX-s model.

#### 4.3.3 TSBA-YOLO

The TSBA-YOLO model is proposed in Ref. [70] to extract global information to find out about tea diseases that are spread across entire areas of leaves and to effectively detect small spots of disease. To increase global receptive field of the model, the transformer's self-attention mechanism is integrated into the backbone of YOLOv5, as depicted in Fig. 9 (c). For effective fusion of multiscale features, BiPEN is employed. A shuffle attention mechanism is added to the neck of YOLOv5 to improve the model's capacity to recognise disease features and express semantic information. The detection head of YOLOv5 is substituted with the proposed adaptively spatial feature fusion (ASFF) detection head, which facilitates the removal of irrelevant information and enables efficient fusion of disease-related details at different scales.

#### 4.3.4 ICM-ODM

To identify disease symptoms severity, the solution proposed in Ref. [71] consists of two modules: image captioning and object detection as shown in Fig. 9 (d). Image captioning is used to generate sentences that contain information about the disease associated with visible symptoms and its severity level. The Image Captioning Model (ICM) uses the pretrained InceptionV3 [54] model as an encoder for feature extraction, and Transformer is used as a decoder for generating caption sentences from features extracted from the encoder. For detecting the infected area and displaying the bounding box around the infected area, YOLOv5 object detection model (ODM) is used. Leaf images are provided simultaneously to both models, and the output image contains a boundary box with sentences that provide information about disease type, symptoms, and degree of damage. The limitation of the proposed model is that ODM performance is very poor.

#### 4.3.5 CTR_YOLOv5n

To enhance the efficiency of maize disease detection, the CTR_YOLOv5n model is proposed in Ref. [72], which embed the Coordinate Attention (CA) and the detection head of Swin Transformer (STR) into YOLOv5n. This modification increases the model's accuracy by 2.8 % compared to the original version. The YOLOv5n object detection model is chosen for its compact size and fast recognition speed. CA mechanism is introduced to the YOLOv5n backbone network to improve focus on smaller pixel blocks, where disease spots only occupy a few pixels in the image. For improving the model's capability in extracting global information, the C3 structure is replaced with the C3STR structure by incorporating the Swin Transformer into a larger detection head, as illustrated in Fig. 9 (e).

#### 4.3.6 YOLOv5s-CA

In order to accurately detect Mummy Berry diseases, [73], introduced the YOLOv5s-CA model. This model incorporates the Coordinated Attention into the backbone of YOLOv5s [41], allowing it to concentrate on visual features related to the disease and amplify the importance of relevant features. This enhancement significantly improves the model's ability to detect diseases, as depicted in Fig. 9 (f). To effectively train the proposed model, the cut-and-paste data augmentation method [74] is employed, which involves creating synthetic images. Additionally, to enhance the localization and bounding box-regression performance of the proposed model when identifying infected areas amidst complex backgrounds, the General Intersection over Union (GIoU) loss function is used.

In object detection, attention mechanisms play a crucial role in considering contextual information for accurate detection. These mechanisms allow the model to focus on relevant regions surrounding an object, helping it gain a better understanding of the object's context and make more informed predictions. By selectively attending to these image regions, the model can emphasize discriminative features, resulting in more precise bounding box predictions. One potential concern with attention mechanisms is their tendency to overly rely on specific regions or features, potentially causing the model to disregard important cues in other parts of the image. To address this, it is essential to strike a balance between the attention mechanism's contribution and other components of the object detection model.

### 4.4 Leveraging attention mechanisms for image classification

Image classification refers to the task of assigning a label or category to an input image. The objective is to create models that can accurately categorize images into predetermined categories. To tackle challenges such as variations in scale, viewpoint, or occlusion, attention mechanisms can be employed. Following are a few models that researchers have recently developed, incorporating attention mechanisms to achieve precise classification of plant diseases.

#### 4.4.1 CD-Mobilenetv3

**Fig. 10:**
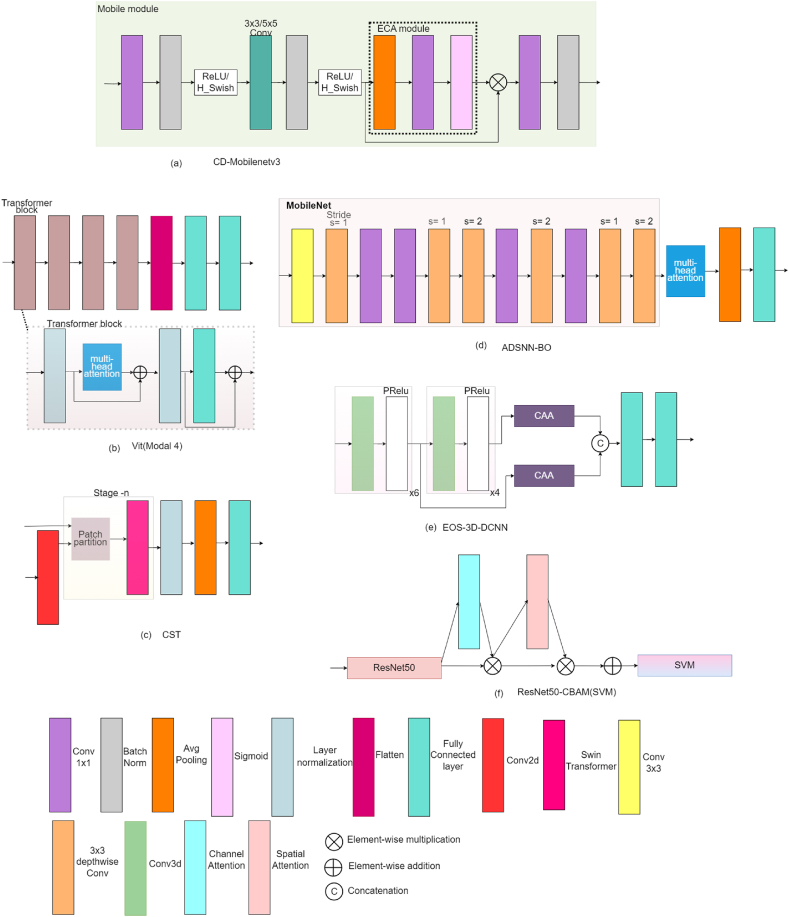
Network design of studies that focus on image classification utilizing attention mechanisms (a) CD- MobileNetV3; (b) Vit (Model 4); (c) CST; (d) ADSNN-BO; (e) EOS-3D-DCNN; (f) ResNet50-CBAM (SVM)

In [75], a CD-Mobilenetv3 model is proposed that uses dilated convolution (Digconv) to broaden the receptive field to ensure that convolution may gather more data and keep its learning attention on samples with special features. To decrease parameters while increasing accuracy, Efficient Channel Attention (ECA) is used in place of the SEs module [9]. Also, to introduce shallow features and to utilize in-depth and local features, cross-layer connections between Mobile modules are introduced as shown in Fig. 10 (a).

#### 4.4.2 ViT

The authors of study [76], suggested a novel vision transformer (ViT) to compare it with CNN for these eight models are proposed that include a combination of four blocks, and each block contains a transformer block, a CNN block, or a combination of these two. The first block contains only CNN blocks, the second block contains only Transformer blocks, the third block contains one CNN block, then a Transformer block follows, and the fourth block contains a Transformer block followed by a CNN block. These eight models were evaluated on three datasets. In every scenario, the ViT model, when trained from the beginning, outperforms CNN or hybrid models in terms of accuracy. Despite having fewer parameters, the proposed ViT model utilizes attention blocks which are relatively slower compared to convolutional blocks. Model 4, shown in Fig. 10 (b), is made entirely of transformer blocks and has the highest recall, f1-score, and precision.

#### 4.4.3 CST

In [77], a Convolutional Swin Transformer (CST) model is introduced, to identify diseases and assess their severity. This model is built upon the Swin Transformer [22]. Three variants of the model (small, large, and base) have been developed, which differ in the way STR blocks are employed in each step and the number of channels utilized. To obtain the image feature map, a convolution block is incorporated within the Swin transformer, allowing the feature map to be inputted into the network for learning, represented in Fig. 10 (c). This modification seeks to improve the model's accuracy and robustness. To address the issues of overfitting and overconfidence, the model incorporates the label smoothing regularization technique. The label smoothing cross-entropy is expressed in Eqs (6), (7):

(6) $$L=-\frac{1}{N}\sum_{i=1}^{N}y_{k}^{LS}\;\ln\left(p_{i}\right)$$

Where,

(7) ykLS=yk(1−α)+αK

Here, Eq. (7) presents a mathematical depiction of label smoothing. In the equation, where $y_{k}$ represents the one-hot encoded representation of a sample i. K denote total number of label categories, α represents a small number (specifically 0.1 in this study), and $p_{i}$ represents the probability that sample i belongs to the positive class.

#### 4.4.4 ADSNN-BO

In [78], a novel model called Attention-based Depthwise Separable Deep Neural Network (ADSNN-BO) is proposed, depicted in Fig. 10 (d). They incorporated an attention layer into the MobileNet model [39], and to optimize the parameters, Bayesian optimization was employed. Using a filter visualization technique, the effectiveness of the suggested approach was tested and compared to existing deep learning model, allowing for detailed analysis and comparison.

#### 4.4.5 EOS-3D-CNN

In [79], a new and innovative approach called the 3D-dense convolutional neural network (3D-DCNN) is introduced for accurately predicting corn disease. They employed the Ebola Optimization Search (EOS) algorithm to determine optimal weights, reduce overall error, and increase accuracy. The VGG16 [36] design is used as the base design for a 3D network. Ten 3D convolution layers are used, which employ a parametric rectified linear unit (PReLU). The model utilizes two Context-Aware Attention (CAA) [80] blocks, depicted in Fig. 10 (e), to capture information at different scales.

#### 4.4.6 ResNet50-CBAM (SVM)

Support vector machines (SVM) is utilized in place of FC network layers to connect to the CNN model in Ref. [81], which also included CBAM for feature extraction as illustrated in Fig. 10 (f). During the second round of training, the base layers of the network are kept “frozen” to minimize trainable parameters after the model has been initially learned from scratch.

Attention mechanisms in image classification have changed the field by improving several aspects of the process. Attention mechanisms enhance object distinction by selectively focusing on particular areas or features within an image, allowing classifiers to more accurately distinguish between objects even in cluttered or complicated scenes. Furthermore, these mechanisms enable more efficient feature selection, enabling models to ignore noisy or irrelevant input and prioritize pertinent data.

Table 1 gives an overview of recent studies that developed techniques that utilized attention mechanisms for creating plant disease identification solutions.

**Table 1:** An overview of recent studies utilizing attention mechanisms for plant disease identification.

Reference	Method	Network Design	Loss Function	Pre-Processing	Framework	Dataset & Accuracy	Performance Metrics & Results	Keywords
[32]	DeepLabV3 + (MobileNetV2 + CBAM + Shuffle Attention + ASPP)	Segmentation	Weighted Loss	Labelme Annotation Tool: To Label the spot at pixel level. Albumentations Data Augmentation Library: For Brightness Adjustments, Cropping, Flipping, Shifting	PyTorch	On Field - Sweetgum Leaf Spot Dataset (SLSD):	Accuracy: 94.5 %	Mixed Attention
[35]	Unet + Wavelet Pooling + Attention Gate Module	Segmentation	Dice Loss	–	–	On Field -CWFID (Crop Weed Field Image Dataset)	IoU: 94.81 %	CNN
[38]	Resnet50 + PAB	Segmentation	–	Random Brightness Level, Random Rotation, Flipping, Hue, Contrast, Saturation Adjustment	PyTorch	Wheat Dataset –Real Field Images	Accuracy: 96.4 %	CNN
[43]	Resnet101 + CBAM + FPN + Segmentation Network (Protonet Branch And The Prediction Head Branch)	Segmentation	Cross-Entropy Loss + Smooth L1 Loss	Random Cropping, Random Contrast, Photometric Distortion, Flipping, Random Rotation	PyTorch	Maize Disease Dataset – Publicly Available	Precision: 98.7 % Mean IoU (mIoU): 84.9 %	Residual Networks
[45]	Resnet50 + FCN	Segmentation	Cross-Entropy Loss	RGB To HSV Colour Space Conversion	PyTorch	Grape Downy Mildew (DM) and Powdery Mildew (PM) Images -Real Field Images	mIoU: 84.%(DM), 74 % (PM)	Hierarchical Multiscale Attention
[46]	Resnet50 + Transformer + MLP + MPH	Segmentation	–	Horizontal and Vertical Flipping, Rotations, Resizing, Normalizations, Segmentation: Copy-Paste Method	PyTorch	Tomato leaf Disease Segmentation Dataset (TDSD)	Accuracy: 96.40 %	Coordinated Attention
[48]	Grapcut + Resnet50 + New-CA, Ghostnet + New-CA	Segmentation	L1 Norm	Random Flipping, Random Rotation, Affine Transformation	PyTorch	Plant Disease Dataset	Top-1 err(%): 12.55 %	CNN With Channel Pruning
[50]	Resnet50 + SE Module	Feature Extraction	–	Rotation, Zooming, Noise Addition, Colour Jitter	PyTorch	Tomato leaf PlantVillage Dataset	Accuracy: 96.81 %	Residual Network
[51]	Inception + RI-Block + CBAM	Feature Extraction	Cross-Entropy Loss	Random Rotation, Random Horizontal and Vertical Offsets, Cross-Cutting Transformation, Random Scaling, Flipping	TensorFlow	Potato, Corn, Tomato -Plantvillage Dataset	Accuracy: 99.55 %	Residual Network
[52]	Pre-Trained CNN (Resnet50, Inceptionv3, VGG19, Mobilenetv2, Efficientnetb0) + CBAM	Feature Extraction	Softmax-Loss	–	Keras	Diamos Plant Dataset – Publicly Available	Accuracy: 86.89 %	Pre-Trained CNN
[55]	Densenet + SE Module + Adabound Optimization Algorithm	Feature Extraction	Cross-Entropy Loss	Random Brightness Level, Viewing Angles, Colours and Horizontal Inversion	PyTorch	Rice Disease Dataset – Publicly Available	Accuracy: 99.4 %	Deep Dense Network
[57]	Dilated CNN + HAM + Logistic Regression	Feature Extraction	Categorical Cross-Entropy Loss	Colour Space Conversion, Bilateral Filtering Data Augmentation: CGAN Segmentation: Otsu's Thresholding	–	Tomato Disease - Plantvillage Dataset	96.6 %	Dilated CNN
[59]	Densenet121 + MFA + SSA	Feature Extraction		Random Clipping, Random Zooming, Horizontal Flipping, Random Rotation	TensorFlow	PDR2018, FGVC8, And PlantDoc Datasets – Publicly Available	Accuracy: 88.32 %(PDR2018), 89.95 % (FGVC8), 89.75 % (PlantDoc)	Multi-Granularity Feature Aggrigation
[60]	Alexnet + Down Sampling Attention Module + Miss Activation Function	Feature Extraction	Binary Cross-Entropy Loss	Random Rotation, Horizontal Flip, Horizontal and Vertical Shift, Random Shear, Zoom	Keras	Corn Disease Dataset- Real Field Images	Accuracy: 99.35 %	Group Convolution
[62]	Inverted Bottleneck Module(With Maxpool) + Shuffle Attention Module	Feature Extraction	Cross-Entropy Loss	Rotation, Cropping, Flipping, Scaling	–	PlantDoc++ Dataset – Publicly Available	Accuracy: 94.6 %	Residual Network
[63]	MS Branch (Atrous Convolution) + DA Branch (SE-Module)	Feature Extraction	Cross-Entropy Loss	–	Paddle	Apple Leaf Disease Dataset – Publicly Available	Accuracy: 96.66 %	Dual Branch CNN
[64]	Efficientnetb6 + CBAM + MSF Module	Feature Extraction	Focal Loss	Image Reverse, Increasing and Decreasing in Brightness level, Horizontal Flipping	PyTorch	Cassava Leaf Disease Dataset- Publicly Available	Accuracy: 88.1 %	Diluted Convolution
[65]	Resnet101 + SE Module + Vit	Feature Extraction	Cross-Entropy Loss	Blurring, Rotation, Noise Addition, Change Brightness, and Darkness level	PyTorch	Python Crawler Tool To Collect Data From Web	Accuracy: 88.34 %	CNN
[66]	Spectral Convolution + Channel Attention Module	Feature Extraction	–	–	–	Fusarium Dataset – Real Field	Accuracy: 82.78 %	Dilated Convolution With Residual Connections
[67]	Yolov5s + CBAM + CA + K-Means	Object Detection	SIoU Loss	Random Rotation, Random Colour Adjustment, Random Brightness Adjustment, Random Contrast Adjustment	PyTorch	Cucumber Root-Knot Nematode Image Dataset - Real Field Images	Mean Average Precision (mAP): 94.8 %	Dual Attention Mechanism
[68]	YOLOX-S + Simam + G-Head	Object Detection	Efficient Intersection over Union (EIoU) loss	LabelImg: To Manually Label Image Data Enhancement - MixUp and Mosaic Method	PyTorch	Fusarium Head Blight Severity Grading Dataset – Real Field Images	mAP: 99.23 %	Ghost Convolution
[70]	Yolov5 + Transformer + Bifpn + Shuffle Attention Module + ASFF	Object Detection	SIoU Loss: For Bounding Box, Binary Cross-Entropy Loss: For Class Probability	Rotation, Colour Dithering, Random Erasing, Image Translation, Mirror Flip	PyTorch	Tea Disease Dataset- Real Field Images	mAP@0.5: 85.35 %	Multiscale Feature Fusion
[71]	Inceptionv3 + Transformer + Yolov5	Object Detection	Sparse Category Cross-Entropy Loss	Linear Contrast, Vertical Flip, Horizontal Flip, Superpixed, Sharpening, Grayscale Conversion, Embossing, Affine transformation	–	Crop Image Dataset – Publicly Available	BLEU Score: 64.96 %(ICM) mAP50: 0.382(ODM)	Pretrained CNN
[72]	Yolov5n + CA + Swin Transformer	Object Detection		Image Annotation Tool: Make Sense AI Augmentation: Changing Colour Brightness, Hue Saturation, Cropping, Scaling, Rotation, Noise Addition, and Mosaic Method	PyTorch	Maize Leaf Dataset -PlantVillage Dataset	mAP: 95.2 %	CNN
[73]	Yolov5s + CA	Object Detection	GIoU Loss	Cut and Paste Technique, Resizing, Rotation	PyTorch	Blueberry Disease Inages-Real-Fields Images	Precision: 96.30 %	Feature Pyramid Network
[75]	MobileNetV3-Large + ECA + Digconv	Image Classification	Bias Loss	Mirror Transformation, Horizontal Flipping, Blurring, Noise, Clipping		Corn Leaf Disease Datasets – Publicly Available	Accuracy: 98.23 %	Dilated Convolution
[76]	Convolution Block + Vision Transformer	Image Classification	–	–	Keras	PlantVillage Dataset, Wheat Rust Classification Dataset, And Rice Leaf Disease Dataset		Hybrid Models
[77]	Convolution + Swin-Transformer	Image Classification	Cross-Entropy Loss with Label Smoothing	Random Horizontal and Vertical Flipping, Bilinear Interpolation - To Adjust the Image Size	PyTorch	Potato Disease Leaf Dataset (PDLD), Tomato Images (Plant Village), Banana Leaf Disease Images (BLDI), And Cucumber Plant Diseases Dataset (CPDD),	Accuracy: 97.5 % (PDLD), 98.2 % (Tomato), 92.2 % (BLDI), 90.9 % (CPDD)	Vision Transformer
[78]	MobileNet + Multi-Head Attention + Bayesian Optimization	Image Classification	–	Otsu's Thresholding, Morphology, Cropping	TensorFlow	Rice Image Dataset – Publicly Available	Accuracy: 94.65 %	Depthwise Convolution
[79]	3DCNN + EOS + CAA	Image Classification	Binary Cross-Entropy Loss	Image Enhancement, Noise Reduction, Scaling, Colour Space Transformation	Keras	Corn Leaf Disease Dataset -Plantvillage Dataset and PlanDoc	Accuracy: 98 %	Feed Forward Neural Network
[81]	Resnet50-CBAM + SVM	Image Classification	First Stage Training - Categorical Cross-Entropy Loss Second Stage Training-Squared Hinge Loss	Data Augmentation - Random Rotation, Random Horizontal Shift, Random Vertical Shift, Horizontal and Vertical Flipping, Zooming, RGB To BGR Conversion Images are Categorized using One-Hot Encoding	–	Tomato Leaf Disease Dataset - Plantvillage Dataset	Accuracy: 97.2 %	Residual Blocks

## 5 Comparative evaluation

**Fig. 11:**
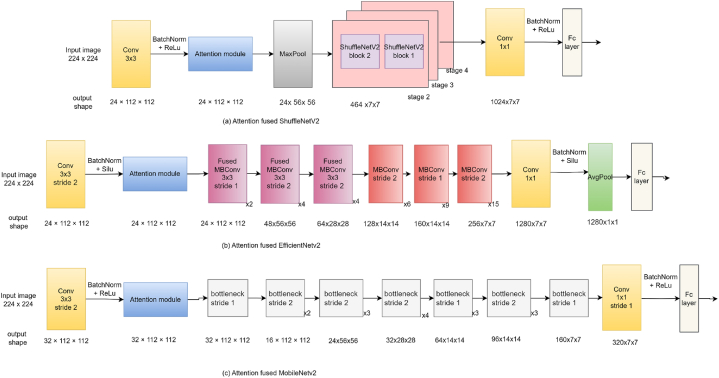
Structure of attention fused model (a) ShuffleNetV2 (b) EfficientNetv2 (c) MobileNetv2

This section presents a comparative study examining the impact of integrating attention modules, such as the SE module [9], CBAM [10], and Shuffle attention [12] modules, into state-of-the-art DL m++odels like ShuffleNetV2 [82], EfficientNetV2 [83], and MobileNetV2 [33]. Our goal is to assess how these modules affect factors such as model performance, efficiency, and parameter count. To achieve this, we've developed attention-fused versions of ShuffleNetV2, EfficientNetV2, and MobileNetV2 models. Initially, an attention module is added after the first convolution layer in each model to enhance the features passed on to subsequent layers. The attention module depicted in Fig. 11 represents the location where various attention mechanisms are applied in the models. Fig. 11 (a) displays the attention-fused ShuffleNetV2 model; the attention-fused EfficientNetV2 model is depicted in Fig. 11 (b); and the attention-fused MobileNetV2 model is presented in Fig. 11 (c).

### 5.1 Material and methods

This comparative study utilizes a publicly available dataset [84] comprising 55,636 images and 5850 augmented images that have been created by performing tasks such as image flipping, scaling transformations, gamma correction, noise injection, rotation, and PCA colour augmentation. The dataset selection was based on its inclusion of images representing 26 diseases across 14 distinct crop species, facilitating comprehensive testing to assess the model's generalizability across diverse classes. The images were captured under controlled conditions with a consistent background and resized to dimensions of 224x224 pixels. The dataset is organized into 39 classes, each corresponding to a specific disease, as outlined in Table 2. To accommodate various deep learning models, additional image pre-processing techniques, such as resizing, are applied. The dataset was divided into three groups: training, validation, and testing, with ratios of 0.8, 0.1, and 0.1, respectively. As a result, 49,188 photos are used for the training set, 6149 for the validation set, and 6149 for the testing set.

**Table 2:** Classification results of attention-fused model considered.

Model	Accuracy	Precision	Recall	F1-Score	Parameters	Impact on accuracy due addition of attention mechanism	Parameters Added
MobileNetV2	99.57 %	99.58 %	99.57 %	99.57 %	2,273,831	–	–
SE_ MobileNetV2	**99.75 %**	**99.75 %**	**99.75 %**	**99.75 %**	2,273,993	+0.18 %	162
CBAM_ MobileNetV2	98.71 %	98.82 %	98.71 %	9.872 %	2,284,247	−0.86 %	10,416
SA_ MobileNetV2	99.70 %	99.70 %	99.70 %	99.7 %	2,273,855	+0.13 %	24
EfficientNetV2	99.70 %	99.72 %	99.70 %	99.69 %	20,227,447	–	–
SE_ EfficientNetV2	99.88 %	99.88 %	99.88 %	99.88 %	20,227,520	-+0.18 %	73
CBAM_ EfficientNetV2	99.38 %	99.42 %	99.38 %	99.38 %	20,237,863	−0.32 %	10,343
SA_ EfficientNetV2	**99.96 %**	**99.96 %**	**99.96 %**	**99.96 %**	20,227,465	+0.26 %	18
ShuffleNetV2	98.67 %	98.33 %	98.67 %	98.44 %	1,293,579	–	
SE_ShuffleNetV2	99.85 %	99.85 %	99.85 %	99.85 %	1,293,652	+1.18 %	73
CBAM_ShuffleNetV2	99.18 %	99.29 %	99.18 %	99.21 %	1,303,995	+0.51 %	10,416
SA_ShuffleNetV2	**99.91 %**	**99.91 %**	**99.91 %**	**99.91 %**	1,293,597	+1.24 %	18

### 5.2 Experiment setup

Most studies in Section 4 and Table 1 used PyTorch, an open-source deep-learning framework popular in research. This study utilized pretrained deep learning models, MobileNetV2, ShuffleNetV2, and EfficientNetV2 models, for comparative analysis. More information and code can be found in Ref. [85]. The experiments were carried out on an NVIDIA GeForce GPU with Driver Version 525.105.17 and CUDA Version 12.0. The GPU's memory capacity was 12,288 MB. The deep learning models used for analysis were pretrained on the ImageNet dataset. The models in our experiment were trained for 15 epochs using a batch size of 32. The ADAM optimizer with an initial learning rate of 0.001 was utilized, and the CrossEntropyLoss function served as the loss function. Additionally, a learning rate decay of 10 % was implemented every 5 epochs to enhance the efficiency of the training process.

### 5.3 Evaluation metrics

Several criteria are taken into account in order to evaluate the models' performance, including accuracy, precision, recall, and F1 score. These indicators are essential for assessing a model's quality. The degree to which the predicted and actual values agree is known as accuracy as shown in Eq. (8). The ratio of true positives to all of the model's positive predictions is known as precision which is given in Eq. (9). Recall measures the ratio of true positives to positive samples in the dataset as depicted in Eq. (10). The F1 score sheds light on the model's capacity to recognise positive samples by combining precision and recall as given in Eq. (11). True Positive (TP), False Positive (FP), True Negative (TN), and False Negative (FN) values are used to calculate evaluation metrics, as explained below:

(8) $$Accuracy=\frac{TP+TN}{TP+FP+TN+FN}$$

(9) $$Precision=\frac{TP}{TP+FP}$$

(10) $$Recall=\frac{TP}{TP+FN}$$

(11) $$F1\;Score=2\frac{Precision*Recall}{Precision+Recall}$$

### 5.4 Results and discussion

The integration of attention processes into deep learning models has resulted in significant increases in model performance across a variety of metrics. Table 2 includes the testing results obtained and compares the performance metrics of various models before and after adding attention techniques such as SENet (SE), CBAM, and Shuffle Attention (SA).

**Fig. 12:**
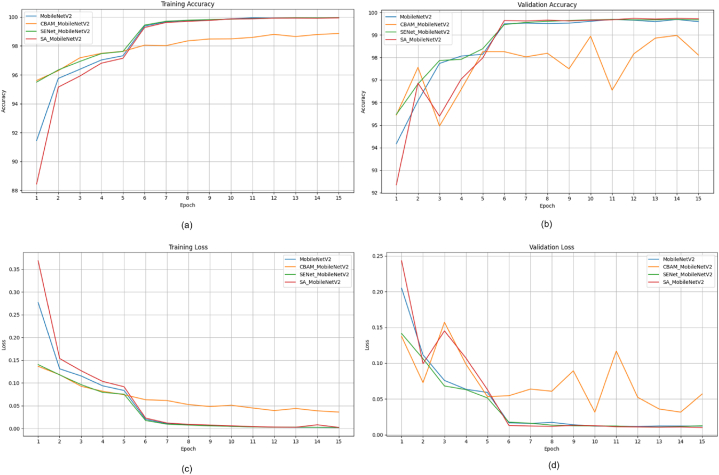
(a) training accuracy; (b) validation accuracy; (c) training loss; and (d) validation loss curves plotted over epochs during the MobileNetV2 and attention-fused MobileNetV2 model training process

Fig. 12 provides a comprehensive overview of the training and validation results of both the standard and attention-fused MobileNetV2 deep learning models, encompassing training accuracy (Fig. 12(a)), validation accuracy (Fig. 12(b)), training loss (Fig. 12(c)), and validation loss (Fig. 12(d)) curves plotted over epochs during the model training process. Based on training and validation data, the MobileNetV2 models' behavior indicates several important trends. Training loss continuously decreases over epochs in all models, suggesting efficient learning processes. Training accuracy increases gradually, with SENet_MobileNetV2 and MobileNetV2 getting close to perfect accuracy. MobileNetV2, CBAM_MobileNetV2, and SENet_MobileNetV2 all show declining validation loss in terms of validation metrics, indicating strong generalization capabilities. As a result, their validation accuracy rates continue to be high, suggesting reliable efficacy on unseen data. SA_MobileNetV2, on the other hand, exhibits variations in both training and validation loss, which may indicate instability or problems with over-fitting. Though SA_MobileNetV2's overall accuracy is not as high as that of other models, it still achieves acceptable validation accuracy. While SA_MobileNetV2 displays certain issues that may call for more research and optimization, MobileNetV2, CBAM_MobileNetV2, and SENet_MobileNetV2 generally show more reliable and efficient behavior in training and validation. As given Table 1, addition of the SE module to MobileNetV2 resulted in a 0.18 % gain in accuracy, precision, recall, and F1-score with only 162 parameters introduced. However, including CBAM resulted in a 0.86 % drop in accuracy while increasing 10,416 parameters. The reason for this decrease could be the placement of CBAM in MobileNetv2. In contrast, SA implementation in MobileNetV2 improved accuracy by 0.13 % with only 24 parameters added.

**Fig. 13:**
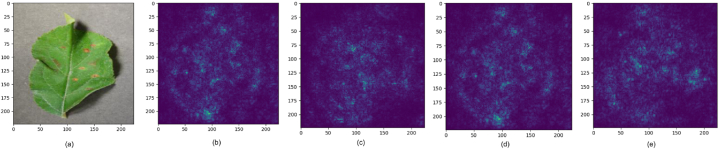
GradCAM visualization of MobileNetV2 for various attention fused models (a) Original Image; (b) MobileNetV2 [33]; (c) CBAM_MobileNetV2; (d) SENet_MobileNetV2; (e) SA_MobileNetV2 model

The focus areas of the models within the images were visualized using GradCAM (Gradient-weighted Class Activation Mapping) following 15 epochs of training. Fig. 13 displays the visual representation of regions within the images where MobileNetV2, CBAM_MobileNetV2, SENet_MobileNetV2, and SA_MobileNetV2 directed their attention. The original image is depicted in Fig. 13(a), while the GradCAM visualization results for MobileNetV2, CBAM_MobileNetV2, SENet_MobileNetV2, and SA_MobileNetV2 are shown in Fig. 13(b), (c), 13(d), and 13(e) respectively. The most discriminative regions that influenced the models' classification decisions are highlighted by this visualization technique. These attention maps can be analyzed to see how each model focused on particular patterns or features of the objects that were important for proper classification. This understanding of the focus areas of the models helps to clarify how they make decisions and shows how well attention mechanisms work to improve object recognition and classification performance.

**Fig. 14:**
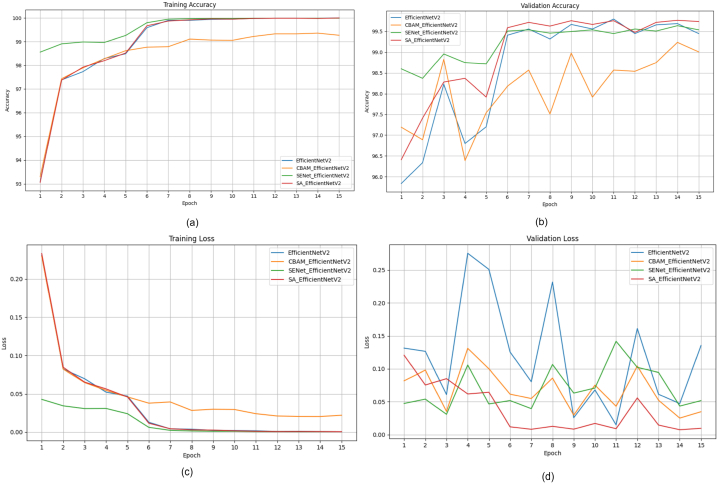
(a) training accuracy; (b) validation accuracy; (c) training loss; and (d) validation loss curves plotted over epochs during the MobileNetV2 and attention-fused EfficientNetV2 model training process

Fig. 14 illustrates the training and validation outcomes of both the standard and attention-enhanced EfficientNetV2 model. This encompasses the depiction of training accuracy (Fig. 14(a)), validation accuracy (Fig. 14(b)), as well as the presentation of training loss (Fig. 14(c)), and validation loss (Fig. 14(d)) curves plotted across epochs throughout the model training phase. As observe, all models as depicted in Fig. 14 (a) shows effective learning during training, SENet_EfficientNetV2 stands out for its strong convergence and performance in both training and validation. However, further investigation may be needed to address the fluctuations observed in validation loss for some models, particularly SENet_EfficientNetV2 and SA_EfficientNetV2. Incorporating the SE module into EfficientNetV2 resulted in a 0.18 % increase in accuracy, along with 73 more parameters. However, CBAM integration resulted in a 0.32 % drop in accuracy, with 10,343 parameters added. In contrast, SA implementation increased accuracy by 0.26 % with only 18 parameters introduced.

**Fig. 15:**
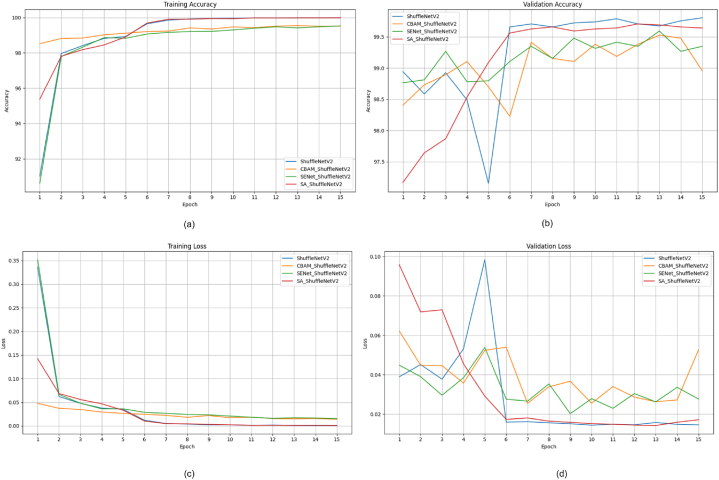
(a) training accuracy; (b) validation accuracy; (c) training loss; and (d) validation loss curves plotted over epochs during the MobileNetV2 and attention-fused ShuffleNetV2 model training process

Fig. 15 demonstrates the training and validation results for both standard and attention-enhanced ShuffleNetV2 deep learning architectures. This includes visualizations of training accuracy (Fig. 15 (a)), validation accuracy (Fig. 15 (b)), along with representations of training loss (Fig. 15 (c)) and validation loss (Fig. 15 (d)) curves plotted across epochs during the model training phase. As evident from Fig. 15 (a), higher training accuracies are achieved by incorporating attention mechanisms, as demonstrated by the CBAM_ShuffleNetV2, SENet_ShuffleNetV2, and SA_ShuffleNetV2 models. This improvement is especially noticeable in later epochs, demonstrating the effectiveness of attention mechanisms in capturing and emphasizing key features during training. The addition of the SE module to ShuffleNetV2 resulted in a remarkable 1.18 % boost in accuracy, along with 73 extra parameters. Similarly, CBAM integration improved accuracy by 0.51 % while adding 10,416 parameters. SA implementation in ShuffleNetV2 resulted in a 1.24 % boost in accuracy while adding only 18 parameters.

**Fig. 16:**
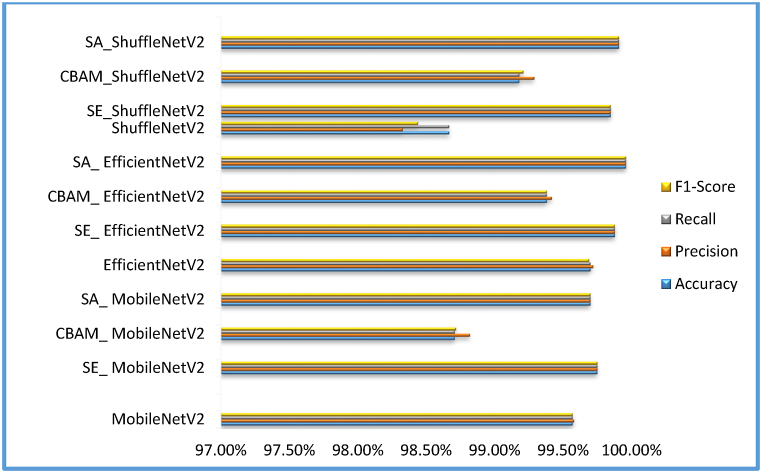
Comparison of model performance on testing data

Overall, these results show that attention processes can help models do better across a number of deep learning architectures when it comes to diagnosing plant diseases. The minor increase in parameters associated with attention mechanism integration shows that the performance benefits outweigh the marginal increase in computational complexity. As a result, attention mechanisms offer a viable option for increasing the efficacy of deep learning models in plant disease recognition. Fig. 16 provides a visual representation of how each model performs in terms of overall accuracy, precision, recall, and F1 score matrices.

After comparative evaluation, it is clear that adding attention modules to deep learning models brings numerous benefits. By leveraging these mechanisms, models can specifically concentrate on relevant data effectively capturing long-range dependencies and contextual information from different positions. However, it is worth mentioning that the incorporation of attention mechanisms could pose problems in terms of increased computational complexity, higher memory requirements, and additional overhead to the overall process.

Despite encountering challenges, attention modules offer significant advantages in advancing deep learning models' capabilities. They are essential in disease identification, serving as a cornerstone for researchers striving for transparency, robustness, and interpretability. The significance of attention is multifaceted. Firstly, it enhances accuracy by enabling models to focus on relevant regions within data, thus improving diagnostic outcomes crucial for reliable disease identification across domains. Secondly, attention mechanisms provide interpretability by generating visual attention maps, offering insights into the model's decision-making process and building trust among users [86]. These attention maps elucidate which regions the model prioritizes, enhancing confidence in its diagnostic abilities. Moreover, attention aids in localizing disease features, facilitating the identification of disease-specific patterns vital for accurate diagnosis, especially in complex disease manifestations. Additionally, integrating attention with other data sources, like sensor data alongside images, holds promise for further improving disease identification accuracy. By leveraging multiple modalities of information, attention-based models can better understand complex disease states, leading to more accurate and comprehensive diagnoses. Furthermore, pre-trained attention mechanisms exhibit transferability, allowing adaptation to new tasks and proving essential for effectively addressing emerging diseases.

### 5.5 Challenges and future scope

Leveraging attention mechanisms offers opportunities and challenges for the field of plant disease identification. Creating a universal attention mechanism that has the ability to handle a variety of tasks without the need for task-specific designs is a significant challenge for future research. In order to streamline model design and enhance model performance across a range of scenarios, it may be possible to combine several attention mechanisms into a single attention block. Currently, different tasks require different attention mechanisms. For instance, while spatial attention is necessary for tasks like object detection and semantic segmentation, channel attention is required for image classification [3,87].

Even though attention mechanisms work well, they are frequently difficult to interpret, making it difficult to determine which areas of the image the model concentrates on in order to identify a disease. In agricultural contexts, developing interpretable attention mechanisms is critical to establishing model validity and fostering trust. Developing techniques to visualize and interpret attention maps could help explain attention mechanisms better and provide important insights into how the model makes decisions. Transparency in the model could be improved by investigating methods for producing clear visualizations that highlight regions of interest that the model identified during the disease identification process.

Furthermore, further study is necessary to completely comprehend the interactions between pre-training and attention mechanisms, even though attention-based models offer task transferability and adaptability to novel inputs. Integrating attention mechanisms with multimodal data sources, such as including sensor data alongside images, could improve disease detection [88,89]. Exploring effective applications of attention across different modalities might lead to more robust identification systems.

Attention mechanisms also find applications in microbiological image analysis. Microscopic images often contain intricate structures, such as bacterial colonies or cellular arrangements. By localizing these features, attention helps identify disease-related patterns. Additionally, to enhance segmentation capabilities, integrating context-aware attention mechanisms, could be beneficial [90]. In histopathology, images reveal spatial patterns linked to tissue morphology, cellular organization, and disease pathology. Utilizing attention mechanisms enables the identification and characterization of these patterns, facilitating the classification of various tissue types and disease states. Studies [91,92] can be benefit from Hierarchical Attention Networks (HANs) [93] that are proposed to attend to features at different levels of abstraction, allowing the model to leverage both local and global contextual information. Furthermore, in a recent study [94], various attention mechanisms like SimAM [69], ECA, and SE modules [9] were employed to direct the model's focus towards relevant features while suppressing irrelevant information. Although these mechanisms were applied separately in each channel, exploring fusion techniques to combine their strengths could lead to more robust feature extraction.

## 6 Conclusion

This study provides a thorough overview of attention mechanisms in the context of plant disease identification, emphasizing their importance in improving feature extraction and model performance. By categorizing and analysing various attention mechanisms, we discovered promising avenues for future research in this area. Investigating attention mechanisms at various stages of disease recognition provides important information for enhancing the interpretability and efficacy of models.

An experiment was conducted on three state-of-the-art deep learning models that incorporate attention layers to evaluate their effectiveness in identifying leaf diseases. The aim was to assess both the efficacy and challenges associated with integrating attention mechanisms into these models.

Our findings reveal that EfficientNetV2 with the SA module surpasses all other models in terms of precision, f1-score, accuracy, and recall. However, when considering factors such as the number of trainable parameters and memory requirements, ShuffleNetV2 with the SA module, which achieves the second highest performance in all metrics, is better suited for mobile and embedded applications. While attention mechanisms enhance the performance of deep learning models, they also introduce computational complexity and require careful hyper parameter tuning. Furthermore, they may have limitations in generalization. Therefore, it is crucial to consider the specific requirements, computational resources, and interpretability needs of the image classification task when deciding whether to incorporate attention mechanisms. The models considered in the comparative study are able to achieve high performance because the dataset used contains images taken against a similar background, which shows little variability. Models performance may degrade when tested in real-field conditions. To promote advancements in this field, future research endeavours should aim to gather extensive and varied datasets that are widely distributed, encouraging exploration and innovation. Future research should prioritize the development of a solution that utilizes attention mechanisms without augmenting computational complexity, which can be achieved by employing shallow networks. Data augmentation techniques, such as GANs, can also be incorporated into the solution as they provide solutions for class imbalance and insufficient dataset problems. Since crop leaves are the subject of the majority of studies, it is important to take other plant components such the stem, flower, and root into account. As global warming intensifies, researchers should focus more on disease recognition in trees to safeguard their protection. In fields like microbiological analysis, attention mechanisms play a crucial role in dissecting complex structures like bacterial colonies, thereby aiding in comprehensive disease assessment. Finally, attention extends its utility beyond disease identification, finding applications in diverse areas such as drug discovery, personalized medicine, and genomics, underscoring its versatility and importance in advancing healthcare research and practice.

## Ethics, approval, and consent to participate

Not applicable.

## Consent to publication

Not applicable.

## Data availability statement

The dataset used in this study is a publicly available dataset that is deposited in the Mendeley Data repository and can be accessed at https://data.mendeley.com/datasets/tywbtsjrjv/1.

## Research support

This research received no external financial or non-financial support.

## Relationship

There are no additional relationships to disclose.

## Patents and intellectual property

There are no patents to disclose.

## Other activities

There are no additional activities to disclose.

## CRediT authorship contribution statement

**Sangeeta Duhan:** Conceptualization, Methodology, Writing – review & editing. **Preeti Gulia:** Conceptualization, Methodology, Writing – review & editing. **Nasib Singh Gill:** Conceptualization, Methodology, Formal analysis, Review and editing. **Piyush Kumar Shukla:** Writing – review & editing. **Surbhi Bhatia Khan:** Methodology, Supervision, Writing – review & editing. **Ahlam Almusharraf:** Supervision, Validation, Writing – review & editing. **Norah Alkhaldi:** Supervison, Visualization, Writing – review & editing.

## Declaration of competing interest

The authors declare that they have no known competing financial interests or personal relationships that could have appeared to influence the work reported in this paper.

## References

[1] Shukla S., Upadhyay D., Mishra A., Jindal T., Shukla K. (2022). Challenges faced by farmers in crops production due to fungal pathogens and their effect on Indian economy. Fungal Biol..

[2] Niu Z., Zhong G., Yu H. (2021, September). A review on the attention mechanism of deep learning. Neurocomputing.

[3] Guo M.H., Xu T.X., Liu J.J., Liu Z.N., Jiang P.T., Mu T.J., Zhang S.H., Martin R.R., Cheng M.M., Hu S.M. (2022, March 15). Attention mechanisms in computer vision: a survey. Computational Visual Media.

[4] Gendy G., He G., Sabor N. (2023, June). Lightweight image super-resolution based on deep learning: state-of-the-art and future directions. Inf. Fusion.

[5] Soydaner D. (2022, May 25). Attention mechanism in neural networks: where it comes and where it goes. Neural Comput. Appl..

[6] Huang J., Gómez-Dans J.L., Huang H., Ma H., Wu Q., Lewis P.E., Xie X. (2019). Assimilation of remote sensing into crop growth models: current status and perspectives. Agric. For. Meteorol..

[7] Abbas A. (2024). Unraveling the threads: a thorough examination of attention mechanisms in deep learning models.

[8] Yang X. (2020, December 1). An overview of the attention mechanisms in computer vision. J. Phys. Conf..

[9] He M., Ren T., Jin Z.D., Deng L., Liu H., Cheng Y.Y., Chang H. (2023). Precise analysis of potassium isotopic composition in plant materials by multi-collector inductively coupled plasma mass spectrometry. Spectrochim. Acta B Atom Spectrosc..

[10] Woo S., Park J., Lee J.Y., Kweon I.S. (2018). CBAM: convolutional block attention module. Computer Vision – ECCV.

[11] Vaswani A., Shazeer N., Parmar N., Uszkoreit J., Jones L., Gomez A.N., Kaiser L., Polosukhin I. (2017, June 12). Attention is all you need. http://arxiv.org/abs/1706.03762.

[12] Yang Q.L.Z.Y.B. (2021, January 30). SA-net: shuffle attention for deep convolutional neural networks. arXiv.org.

[13] Xu K., Ba J., Kiros R., Cho K., Courville A., Salakhutdinov R., Zemel R., Bengio Y. (2015, February 10). Show, attend and tell: neural image caption generation with visual attention. arXiv.org.

[14] Hou Q., Zhou D., Feng J. (2021, March 4). Coordinate attention for efficient mobile network design. http://arxiv.org/abs/2103.02907.

[15] Fu J., Liu J., Tian H., Li Y., Bao Y., Fang Z., Lu H. (2018, September 9). Dual attention network for scene segmentation. arXiv.org.

[16] Gheini M., Ren X., May J. (2021, April 18). Cross-attention is all you need: adapting pretrained transformers for machine translation. arXiv.org.

[17] Chen C.F.R., Fan Q., Panda R. (2021, October). 2021 IEEE/CVF International Conference on Computer Vision (ICCV).

[18] Petit O., Thome N., Rambour C., Soler L. (2021, March 10). U-net transformer: self and cross attention for medical image segmentation. http://arxiv.org/abs/2103.06104.

[19] Kortschak H. (2022, February 19). Attention and transformer models - towards data science. Medium.

[20] Dosovitskiy A., Beyer L., Kolesnikov A., Weissenborn D., Zhai X., Unterthiner T., Dehghani M., Minderer M., Heigold G., Gelly S., Uszkoreit J., Houlsby N. (2020, October 22). An image is worth 16x16 words: transformers for image recognition at scale. http://arxiv.org/abs/2010.11929.

[21] Touvron H., Cord M., Douze M., Massa F., Sablayrolles A., Jégou H. (2020, December 23). Training data-efficient image transformers & distillation through attention.

[22] Liu Z., Lin Y., Cao Y., Hu H., Wei Y., Zhang Z., Lin S., Guo B. (2021, March 25). Swin transformer: hierarchical vision transformer using shifted windows. arXiv.org.

[23] Heo B., Yun S., Han D., Chun S., Choe J., Oh S.J. (2021, March 30). Rethinking spatial dimensions of vision transformers. arXiv.org.

[24] Carion N., Massa F., Synnaeve G., Kirillov A., Zagoruyko S. (2020, May 26). End-to-End object detection with transformers. arXiv.org.

[25] Luong M.T., Pham H., Manning C.D. (2015, August 17). Effective approaches to attention-based neural machine translation. arXiv.org.

[26] Lin T.Y., Dollár P., Girshick R., He K., Hariharan B., Belongie S. (2016, December 9). Feature pyramid networks for object detection. arXiv.org.

[27] Zhao H., Shi J., Qi X., Wang X., Jia J. (2016, December 4). Pyramid scene parsing network.

[28] Wang X., Girshick R., Gupta A., He K. (2017, November 21). Non-local neural networks.

[29] Yu F., Wang D., Shelhamer E., Darrell T. (2017, July 20). Deep layer aggregation. http://arxiv.org/abs/1707.06484.

[30] Li Y., Chen Y., Wang N., Zhang Z.X. (2019, October). 2019 IEEE/CVF International Conference on Computer Vision (ICCV).

[31] Liu S., Qi L., Qin H., Shi J., Jia J. (2018, March 5). Path aggregation network for instance segmentation. arXiv.org. http://arxiv.org/abs/1803.01534.

[32] Cai M., Yi X., Wang G., Mo L., Wu P., Mwanza C., Kapula K.E. (2022, December 8). Image segmentation method for sweetgum leaf spots based on an improved DeeplabV3+ network. Forests.

[33] Sandler M., Howard A., Zhu M., Zhmoginov A., Chen L.C. (2018, June). 2018 IEEE/CVF Conference on Computer Vision and Pattern Recognition.

[34] Chen L.C., Papandreou G., Schroff F., Adam H. (2017, June 17). Rethinking atrous convolution for semantic image segmentation. arXiv.org.

[35] Banu A.S., Deivalakshmi S. (2022, December 12). AWUNet: leaf area segmentation based on attention gate and wavelet pooling mechanism. Signal, Image and Video Processing.

[36] Simonyan K., Zisserman A. (2014, September 4). Very deep convolutional networks for large-scale image recognition.

[37] He K., Zhang X., Ren S., Sun J. (2016, June). 2016 IEEE Conference on Computer Vision and Pattern Recognition (CVPR).

[38] Cheng S., Cheng H., Yang R., Zhou J., Li Z., Shi B., Lee M., Ma Q. (2023, March 6). A high performance wheat disease detection based on position information. Plants.

[39] Howard A.G., Zhu M., Chen B., Kalenichenko D., Wang W., Weyand T., Andreetto M., Adam H. (2017, April 17). MobileNets: efficient convolutional neural networks for mobile vision applications. http://arxiv.org/abs/1704.04861.

[40] Redmon J., Farhadi A. (2018, April 8). YOLOv3: an incremental improvement. http://arxiv.org/abs/1804.02767.

[41] ultralytics/yolov5 (2022, November 23).

[42] He K., Gkioxari G., Dollár P., Girshick R. (2017, March 20). Mask R-CNN. http://arxiv.org/abs/1703.06870.

[43] Huang M., Xu G., Li J., Huang J. (2021, December 2). A method for segmenting disease lesions of maize leaves in real time using attention YOLACT++. Agriculture.

[44] Tao A., Sapra K., Catanzaro B. (2020, May 21). Hierarchical multi-scale attention for semantic segmentation. arXiv.org.

[45] Liu E., Gold K.M., Combs D., Cadle-Davidson L., Jiang Y. (2022, September 9). Deep semantic segmentation for the quantification of grape foliar diseases in the vineyard. Front. Plant Sci..

[46] Wu J., Wen C., Chen H., Ma Z., Zhang T., Su H., Yang C. (2022, August 26). DS-DETR: a model for tomato leaf disease segmentation and damage evaluation. Agronomy.

[47] Gao P., Zheng M., Wang X., Dai J., Li H. (2021, October). 2021 IEEE/CVF International Conference on Computer Vision (ICCV).

[48] Qi F., Wang Y., Tang Z. (2022, June 6). Lightweight plant disease classification combining GrabCut algorithm, new coordinate attention, and Channel pruning. Neural Process. Lett..

[49] Han K., Wang Y., Tian Q., Guo J., Xu C., Xu C. (2019, November 27). GhostNet: more features from cheap operations. http://arxiv.org/abs/1911.11907.

[50] Zhao S., Peng Y., Liu J., Wu S. (2021, July 11). Tomato leaf disease diagnosis based on improved convolution neural network by attention module. Agriculture.

[51] Zhao Y., Sun C., Xu X., Chen J. (2022, February). RIC-Net: a plant disease classification model based on the fusion of Inception and residual structure and embedded attention mechanism. Comput. Electron. Agric..

[52] Alirezazadeh P., Schirrmann M., Stolzenburg F. (2022, December 16). Improving deep learning-based plant disease classification with attention mechanism. Gesunde Pflanz..

[53] Tan M., Le Q. (2019, May 28). EfficientNet: rethinking model scaling for convolutional neural networks. http://arxiv.org/abs/1905.11946.

[54] Szegedy C., Vanhoucke V., Ioffe S., Shlens J., Wojna Z. (2016, June). 2016 IEEE Conference on Computer Vision and Pattern Recognition (CVPR).

[55] Jiang M., Feng C., Fang X., Huang Q., Zhang C., Shi X. (2023, January 18). Rice disease identification method based on attention mechanism and deep dense network. Electronics.

[56] Huang G., Liu Z., Van Der Maaten L., Weinberger K.Q. (2017, July). 2017 IEEE Conference on Computer Vision and Pattern Recognition (CVPR).

[57] Islam M.S., Sultana S., Farid F.A., Islam M.N., Rashid M., Bari B.S., Hashim N., Husen M.N. (2022, August 14). Multimodal hybrid deep learning approach to detect tomato leaf disease using attention based dilated convolution feature extractor with logistic regression classification. Sensors.

[58] Mirza M., Osindero S. (2014, November 6). Conditional generative adversarial nets. http://arxiv.org/abs/1411.1784.

[59] Zuo X., Chu J., Shen J., Sun J. (2022, September 19). Multi-granularity feature aggregation with self-attention and spatial reasoning for fine-grained crop disease classification. Agriculture.

[60] Wang Y., Tao J., Gao H. (2022, September 18). Corn disease recognition based on attention mechanism network. Axioms.

[61] Krizhevsky A., Sutskever I., Hinton G.E. (2017, May 24). ImageNet classification with deep convolutional neural networks. Commun. ACM.

[62] Zhang R., Wang Y., Jiang P., Peng J., Chen H. (2023, March 29). IBSA_Net: a network for tomato leaf disease identification based on transfer learning with small samples. Appl. Sci..

[63] Perveen K., Kumar S., Kansal S., Soni M., Alshaikh N.A., Batool S., Khanam M.N., Osei B. (2023). Multidimensional attention-based CNN model for identifying apple leaf disease. J. Food Qual..

[64] Liu M., Liang H., Hou M. (2022, December 22). Research on cassava disease classification using the multi-scale fusion model based on EfficientNet and attention mechanism. Front. Plant Sci..

[65] Zeng Q., Niu L., Wang S., Ni W. (2022, December 19). SEViT: a large-scale and fine-grained plant disease classification model based on transformer and attention convolution. Multimed. Syst..

[66] Khotimah W.N., Boussaid F., Sohel F., Xu L., Edwards D., Jin X., Bennamoun M. (2022, August 30). SC-CAN: spectral convolution and Channel Attention network for wheat stress classification. Rem. Sens..

[67] Wang C., Sun S., Zhao C., Mao Z., Wu H., Teng G. (2022, October 19). A detection model for cucumber root-knot nematodes based on modified YOLOv5-CMS. Agronomy.

[68] Mao R., Wang Z., Li F., Zhou J., Chen Y., Hu X. (2023, January 13). GSEYOLOX-S: an improved lightweight network for identifying the severity of wheat Fusarium head blight. Agronomy.

[69] Yang, L., Zhang, R.-Y., Li, L., & Xie, X. (n.d.). SimAM: A Simple, Parameter-free Attention Module for Convolutional Neural Networks..

[70] Lin J., Bai D., Xu R., Lin H. (2023, March 20). TSBA-YOLO: an improved tea diseases detection model based on attention mechanisms and feature fusion. Forests.

[71] Lee D.I., Lee J.H., Jang S.H., Oh S.J., Doo I.C. (2023, February 28). Crop disease diagnosis with deep learning-based image captioning and object detection. Appl. Sci..

[72] Ma L., Yu Q., Yu H., Zhang J. (2023, February 11). Maize leaf disease identification based on YOLOv5n algorithm incorporating attention mechanism. Agronomy.

[73] Obsie E.Y., Qu H., Zhang Y.J., Annis S., Drummond F. (2022, December 27). Yolov5s-CA: an improved Yolov5 based on the attention mechanism for Mummy Berry disease detection. Agriculture.

[74] Dwibedi D., Misra I., Hebert M. (2017, August 4). Cut, paste and learn: surprisingly easy synthesis for instance detection. arXiv.org.

[75] Bi C., Xu S., Hu N., Zhang S., Zhu Z., Yu H. (2023, January 18). Identification method of corn leaf disease based on improved Mobilenetv3 model. Agronomy.

[76] Borhani Y., Khoramdel J., Najafi E. (2022, July 7). A deep learning based approach for automated plant disease classification using vision transformer. Sci. Rep..

[77] Guo Y., Lan Y., Chen X. (2022, November). CST: convolutional Swin Transformer for detecting the degree and types of plant diseases. Comput. Electron. Agric..

[78] Wang Y., Wang H., Peng Z. (2021, September). Rice diseases detection and classification using attention based neural network and bayesian optimization. Expert Syst. Appl..

[79] Ashwini C., Sellam V. (2023, March 28). EOS-3D-DCNN: Ebola optimization search-based 3D-dense convolutional neural network for corn leaf disease prediction. Neural Comput. Appl..

[80] Yang B., Li J., Wong D., Chao L.S., Wang X., Tu Z. (2019, February 15). Context-aware self-attention networks. http://arxiv.org/abs/1902.05766.

[81] Altalak M., Uddin M.A., Alajmi A., Rizg A. (2022, August 16). A hybrid approach for the detection and classification of tomato leaf diseases. Appl. Sci..

[82] Ma N., Zhang X., Zheng H.T., Sun J. (2018, July 30). ShuffleNet V2: practical guidelines for efficient CNN architecture design. http://arxiv.org/abs/1807.11164.

[83] Tan M., Le Q. (2021, April 1). EfficientNetV2: smaller models and faster training. http://arxiv.org/abs/2104.00298.

[84] (2019, June). Identification of plant leaf diseases using a nine-layer deep convolutional neural network. Comput. Electr. Eng..

[85] Du K., Huang J., Wang W., Zeng Y., Li X., Zhao F. (2024). Monitoring low-temperature stress in winter wheat using TROPOMI solar-induced chlorophyll fluorescence. IEEE Trans. Geosci. Rem. Sens..

[86] Thakur P.S., Khanna P., Sheorey T., Ojha A. (2022, July 16). Explainable vision transformer enabled convolutional neural network for plant disease identification: PlantXViT.

[87] Huang J., Gómez-Dans J.L., Huang H., Ma H., Wu Q., Lewis P.E., Xie X. (2019). Assimilation of remote sensing into crop growth models: current status and perspectives. Agric. For. Meteorol..

[88] Liu Y., Li H., Hu C., Luo S., Luo Y., Chen C.W. (2024). Learning to aggregate multi-scale context for instance segmentation in remote sensing images. IEEE Transact. Neural Networks Learn. Syst..

[89] Huang L., Wang Z., Fu X. (2023, June 1). Pedestrian detection using RetinaNet with multi-branch structure and double pooling attention mechanism. Multimed. Tool. Appl..

[90] Zhang J., Li C., Kosov S., Grzegorzek M., Shirahama K., Jiang T., Sun C., Li Z., Li H. (2021, July). LCU-Net: a novel low-cost U-Net for environmental microorganism image segmentation. Pattern Recogn..

[91] Kulwa F., Li C., Zhang J., Shirahama K., Kosov S., Zhao X., Jiang T., Grzegorzek M. (2022, March 7). A new pairwise deep learning feature for environmental microorganism image analysis. Environ. Sci. Pollut. Control Ser..

[92] Chen H., Li C., Wang G., Li X., Mamunur Rahaman M., Sun H., Hu W., Li Y., Liu W., Sun C., Ai S., Grzegorzek M. (2022, October). GasHis-Transformer: a multi-scale visual transformer approach for gastric histopathological image detection. Pattern Recogn..

[93] Liu W., Ding L., Su H. (2021, July 13). HANA: hierarchical attention network assembling for semantic segmentation. Cognitive Computation.

[94] Chen H., Li C., Li X., Rahaman M.M., Hu W., Li Y., Liu W., Sun C., Sun H., Huang X., Grzegorzek M. (2022, April). IL-MCAM: an interactive learning and multi-channel attention mechanism-based weakly supervised colorectal histopathology image classification approach. Comput. Biol. Med..

